# Supplementary material for: Room-temperature optically detected magnetic resonance of single defects in hexagonal boron nitride
Source: Nat Commun. 2022 Feb 1;13:618. doi: 10.1038/s41467-022-28169-z (PMC8807746; doi:10.1038/s41467-022-28169-z)
Supplement: Supplementary file 1 — supplementary information [file 41467_2022_28169_MOESM1_ESM.pdf]

Supplementary Information

Room-temperature optically detected magnetic resonance of single defects in hexagonal boron nitride.

Hannah L. Stern[1*†], Qiushi Gu[1*], John Jarman[1*], Simone Eizagirre Barker[1], Noah Mendelson[2], Dipankar Chugh[3], Sam Schott[1], Hoe H. Tan[3], Henning Sirringhaus[1], Igor Aharonovich[2] and Mete Atatüre[1†].

[1] *Cavendish Laboratory, University of Cambridge, J.J. Thomson Ave., Cambridge, CB3 0HE, United Kingdom.*

[2] *ARC Centre of Excellence for Transformative Meta-Optical Systems, Faculty of Science, University of Technology Sydney, New South Wales, Australia.*

[3] *ARC Centre of Excellence for Transformative Meta-Optical Systems, Research School of Physics and Engineering, The Australian National University, Canberra, Australian Capital Territory, Australia.*

* These authors contributed equally

[†]Corresponding authors: H.L.S. (hs536@cam.ac.uk) and M.A. (ma424@cam.ac.uk).

Contents

1) Material characterisation
   a) Confocal measurements
   b) Laser power saturation of integrated-PL intensity
   c) Second-order autocorrelation measurements
   d) Kinetic Model
      i) ODMR contrast as a function of laser power
      ii) Positive and negative ODMR contrast
      iii) Presence or absence of ODMR

2) ODMR of single defects
   a) Determination of saturation conditions: microwave power.
   b) Determination of saturation conditions: laser power
   c) Analysis of functions fit to model ODMR lineshape
   d) PL spectra variation of single defects
   e) Summary of all ODMR-active defects

3) ODMR of high-density ensemble
   a) G-factor calibration
   b) ODMR lineshape of high-density ensemble

4) EPR of high-density ensemble

5) Spin Hamiltonian for S=1
   a) Angular-dependent ODMR measurements

6) References

1. Material characterisation

a) Confocal measurements

Photoluminescence measurements were performed on a room-temperature home-built confocal setup described in Methods. Figure 1a shows an example 8x6 µm confocal image obtained by integrated-PL intensity under 100-μW 532-nm laser excitation. Panel b is a high-resolution 1.5x1.5 µm confocal image of defect A.

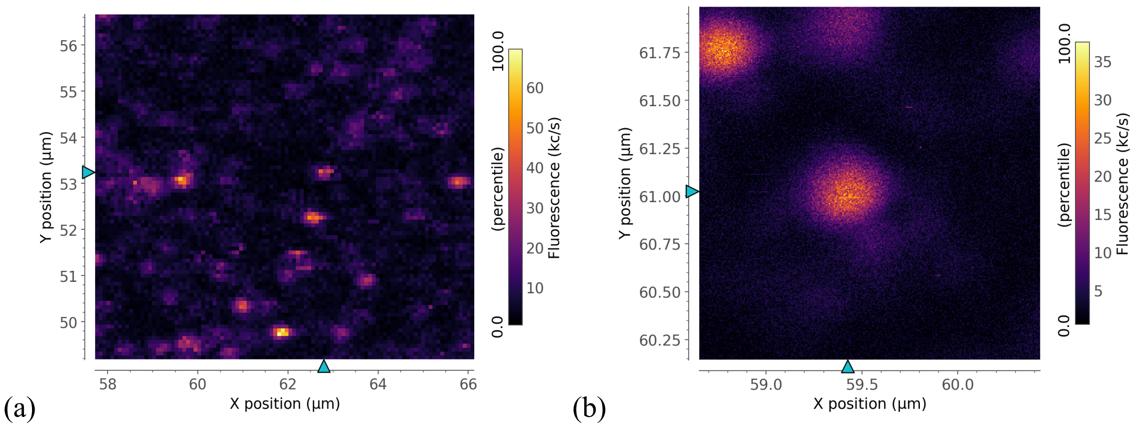

(a) (b)

Supplementary Figure 1: Confocal maps of the low-density hBN sample. (a) large-area image displaying multiple isolated defects, (b) high-resolution image of defect A.

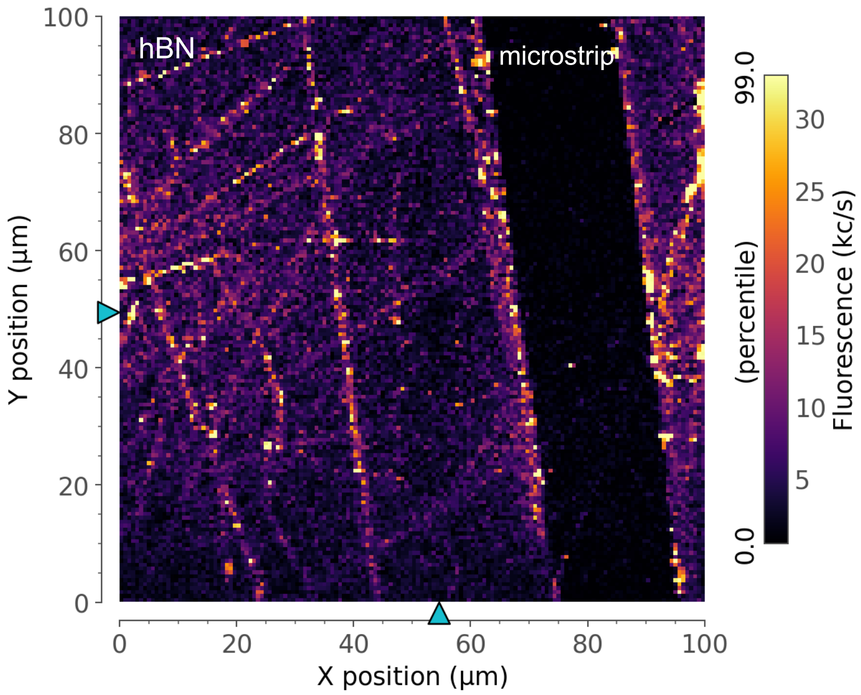

Supplementary Figure 2: Confocal maps of the low-density hBN sample. The bright areas coincide with wrinkles in the material.

b) Laser power saturation of integrated-PL intensity

Photoluminescence saturation measurements as a function of laser power were recorded with 532-nm excitation up to 350-μW power and the PL was collected through a 550-nm long pass filter.

Data set is fit to the function:

$$I = \frac{I_{\text{sat}} P}{P + P_{\text{sat}}} \quad \text{(Eq. 1)}$$

Where I is the PL intensity, P is the laser power, I_{sat} is the PL intensity (cps) at saturation and P_{sat} is the saturation laser power. We find that there is significant variation in these parameters across the defects we measure (see Supplementary Table 3).

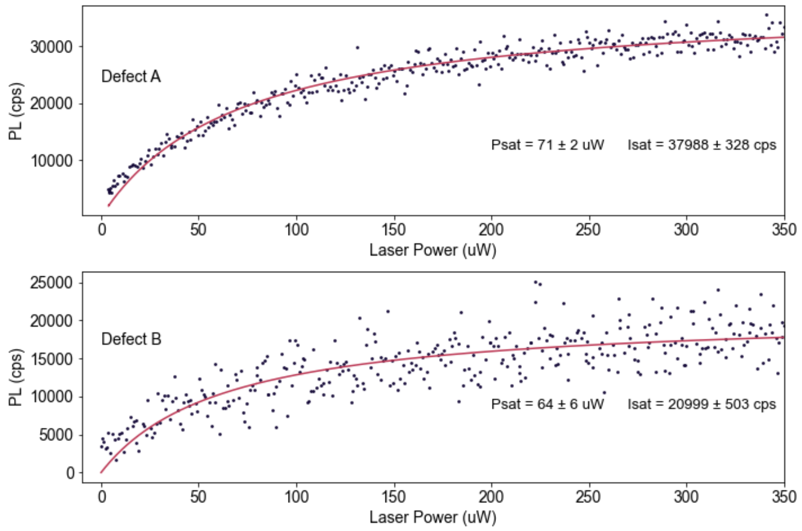

Supplementary Figure 3: Integrated PL intensity as a function of the excitation laser power for defects A (top) and B (bottom) in the main text.

c) Second-order intensity-correlation, $g^{(2)}(\tau)$, measurements.

Second-order intensity-correlation ($g^{(2)}(\tau)$) measurements were performed by measuring the photon arrival time at two detectors. The recorded arrival times at detector 1 were compared with the arrival times recorded at detector 2, to form an autocorrelation that extends to 1 millisecond separation time. The data presented in the main text Figure 1c and Supplementary Figure 4 for defect A and Supplementary Figure 5-6 for defect B and C represent autocorrelation of over 40 million photon detection events recorded for each APD channel.

For all $g^{(2)}(\tau)$ measurements, the time stamps from -50 ns to 50 ns are binned in linear-scale time bins, whereas the data at longer time delays are binned in logarithmic-scale bins, to aid visualisation. To clarify this, below we showed the linear time region (-50 to 50 ns) as well as the full linear-log plot (-1 ms to 1 ms) for autocorrelation measurements of the defects. The autocorrelation data is recorded with a time tagger with a time resolution of 81 ps, which corresponds to the 'hardware' bin size. The hardware bins are then averaged to process and present the data. In the main text we chose to present the data where ten adjacent bins are averaged (i.e., with a bin size of 810 ps in the x axis), as this gives a compromise between lowering the scatter in the data to aid fitting, but not completely over smoothing the data at $\boldsymbol{\tau} = 0$.

We fit the data to both a tri-exponential and bi-exponential function (Equation 2 and 3) but find the autocorrelation data for the three defects we present is best fit to a tri-exponential function (Equation 2). We report the non-background corrected extracted timescales for both in Table 1, below.

Fit functions used for $g^{(2)}(\tau)$ measurements:

$$g^{(2)}(\tau) = y_0 - ae^{(-|\tau-t0|/\tau 1)} + be^{(-|\tau-t0|/\tau 2)} + ce^{(-|\tau-t0|/\tau 3)} \qquad \text{(Eq. 2)}$$
$$g^{(2)}(\tau) = y_0 - ae^{(-|\tau-t0|/\tau 1)} + be^{(-|\tau-t0|/\tau 2)} \qquad \text{(Eq. 3)}$$

Supplementary Table 1: Fit parameters extracted from applying the above functions to the $g^{(2)}(\tau)$ data shown in Supplementary Figures. 4, 5 and 6.

	Defect A (Tri-exponential fit)	Defect A (Bi-exponential fit)	Defect B (Tri-exponential fit)	Defect B (Bi-exponential fit)	Defect C (Tri-exponential fit)	Defect C (Bi-exponential fit)
$g^{(2)}(0)$	0.34 ± 0.03	0.33 ± 0.06	0.35 ± 0.06	0.28 ± 0.09	0.25 ± 0.01	0.22 ± 0.02
y_0	36.5 ± 0.1	36.9 ± 0.1	69.4 ± 0.4	72 ± 0.5	35 ± 0.05	35 ± 0.1
a	34.4 ± 1.4	34.5 ± 1.6	146 ± 2	145 ± 3	42 ± 1.3	42 ± 1.5
$\tau 1$ (ns)	1.6 ± 0.1	1.6 ± 0.2	4.9 ± 0.1	4.5 ± 0.15	2.43 ± 0.09	2.24 ± 0.1
b	5.0 ± 0.4	9.6 ± 0.1	15.8± 0.4	95 ± 0.5	4.9 ± 0.04	15 ± 0.07
$\tau 2$ (ns)	2.5e4 ± 3.7e3	8.1e3	4.6e3 ± 290	8.7e4 ± 1.4 e3	9.40e2 ± 1.0e2	4.7e3 ± 81
c	5.3 ± 0.5		85.6 ± 0.4		11 ± 0.4	
$\tau 3$ (ns)	2.8e3 ± 383		1.2e5 ± 2e3		7.9e3 ± 3.7e2	

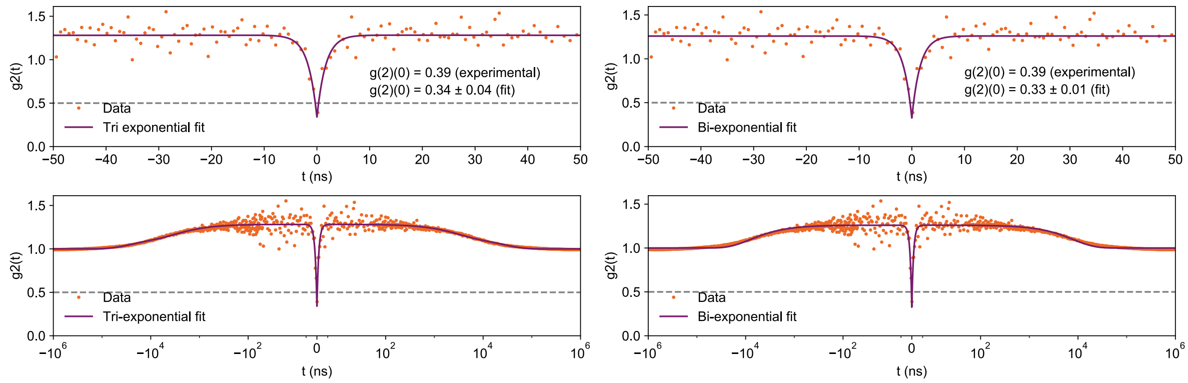

Supplementary Figure 4: $g^{(2)}(\tau)$ measurement for defect A fit to a tri-exponential (left) and bi-exponential (right) function. The top panels show a zoomed in time range of -50 to 50 ns, the bottom panels show the same data from -1 ms to 1 ms. Measurement carried out at $1.4P_{sat}^{optical}$ excitation.

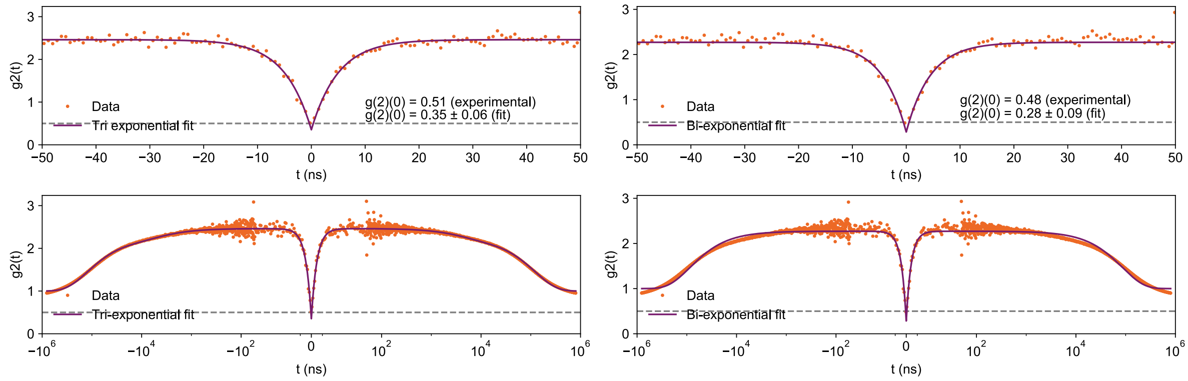

Supplementary Figure 5: $g^{(2)}(\tau)$ measurement for defect B fit to a tri-exponential (left) and bi-exponential (right) function. The top panels show a zoomed in time range of -50 to 50 ns, the bottom panels show the same data from -1 ms to 1 ms. Measurement carried out at $1.6P_{sat}^{optical}$ laser power.

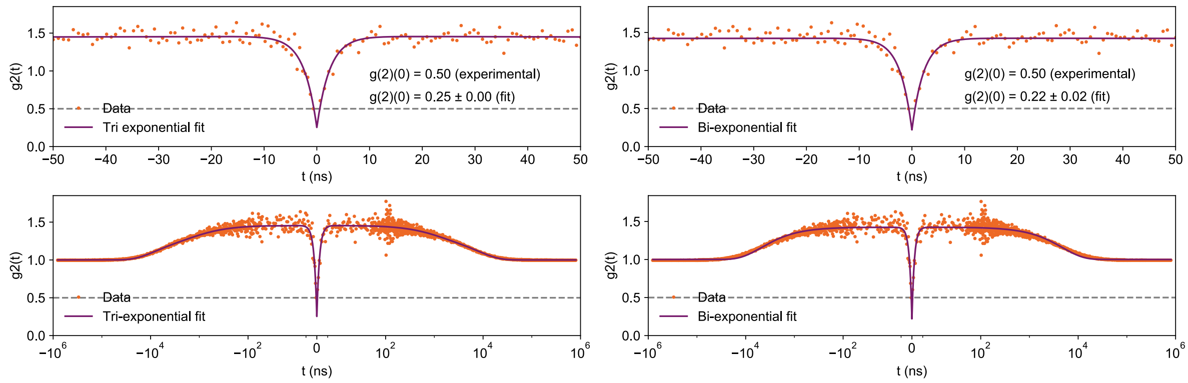

Supplementary Figure 6: $g^{(2)}(\tau)$ measurement for defect C fit to a tri-exponential (left) and bi-exponential (right) function. The top panels show a zoomed in time range of -50 to 50 ns, the bottom panels show the same data from -1 ms to 1 ms. Measurements carried out at $0.1P_{sat}^{optical}$ laser power.

Additional $g^{(2)}(\tau)$ Measurements for ODMR Active Defects:

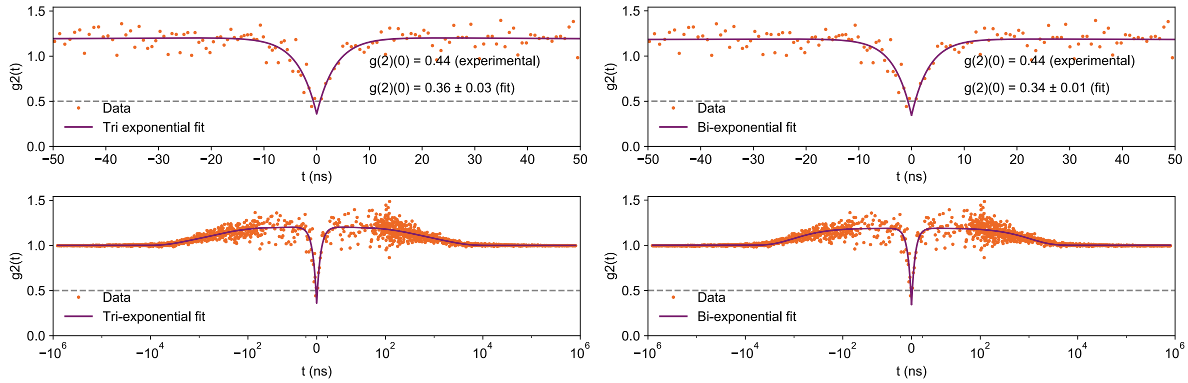

Supplementary Figure 7: $g^{(2)}(\tau)$ measurement for defect D fit to a tri-exponential (left) and bi-exponential (right) function. The top panels show a zoomed in time range of -50 to 50 ns, the bottom panels show the same data from -1 ms to 1 ms. Measurements carried out at $0.3P_{sat}^{optical}$ laser power.

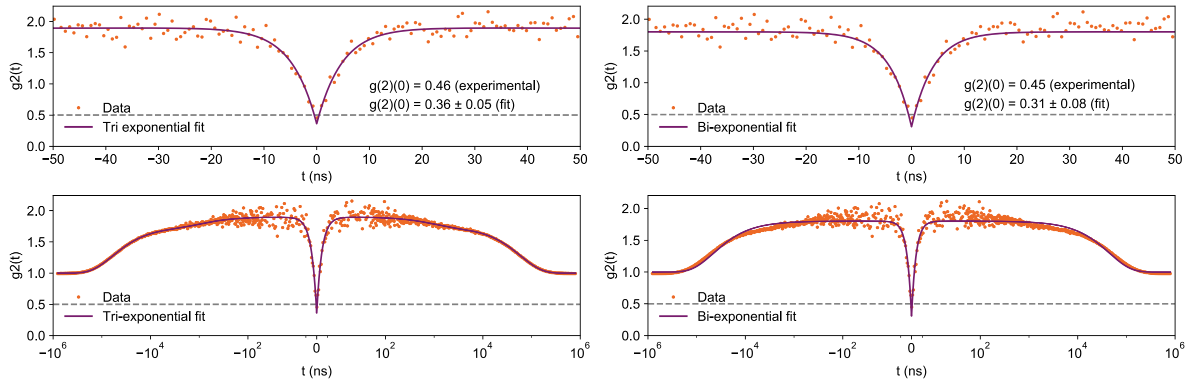

Supplementary Figure 8: $g^{(2)}(\tau)$ measurement for defect E fit to a tri-exponential (left) and bi-exponential (right) function. The top panels show a zoomed in time range of -50 to 50 ns, the bottom panels show the same data from -1 ms to 1 ms. Measurements carried out at $0.12P_{sat}^{optical}$ laser power.

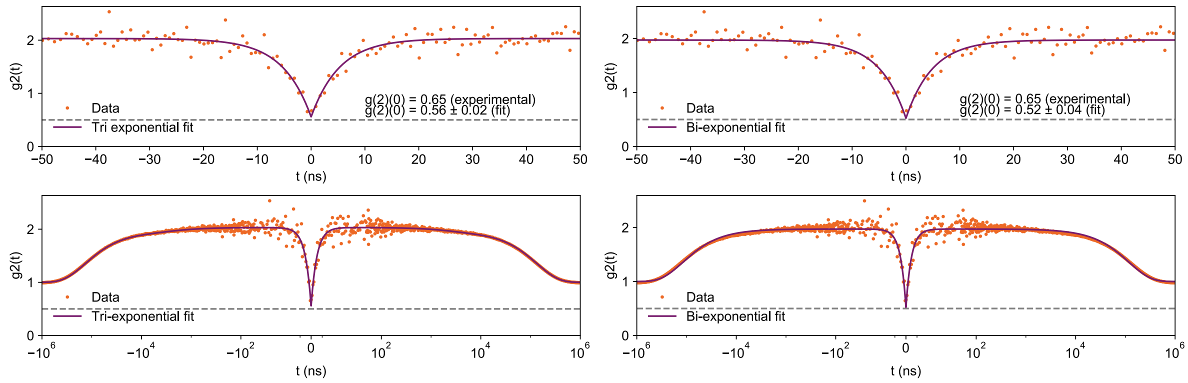

Supplementary Figure 9: $g^{(2)}(\tau)$ measurement for defect K fit to a tri-exponential (left) and bi-exponential (right) function. The top panels show a zoomed in time range of -50 to 50 ns, the bottom panels show the same data from -1 ms to 1 ms. Measurements carried out at $0.12P_{sat}^{optical}$ laser power.

$g^{(2)}(\tau)$ Measurements for Non-ODMR Active Defects

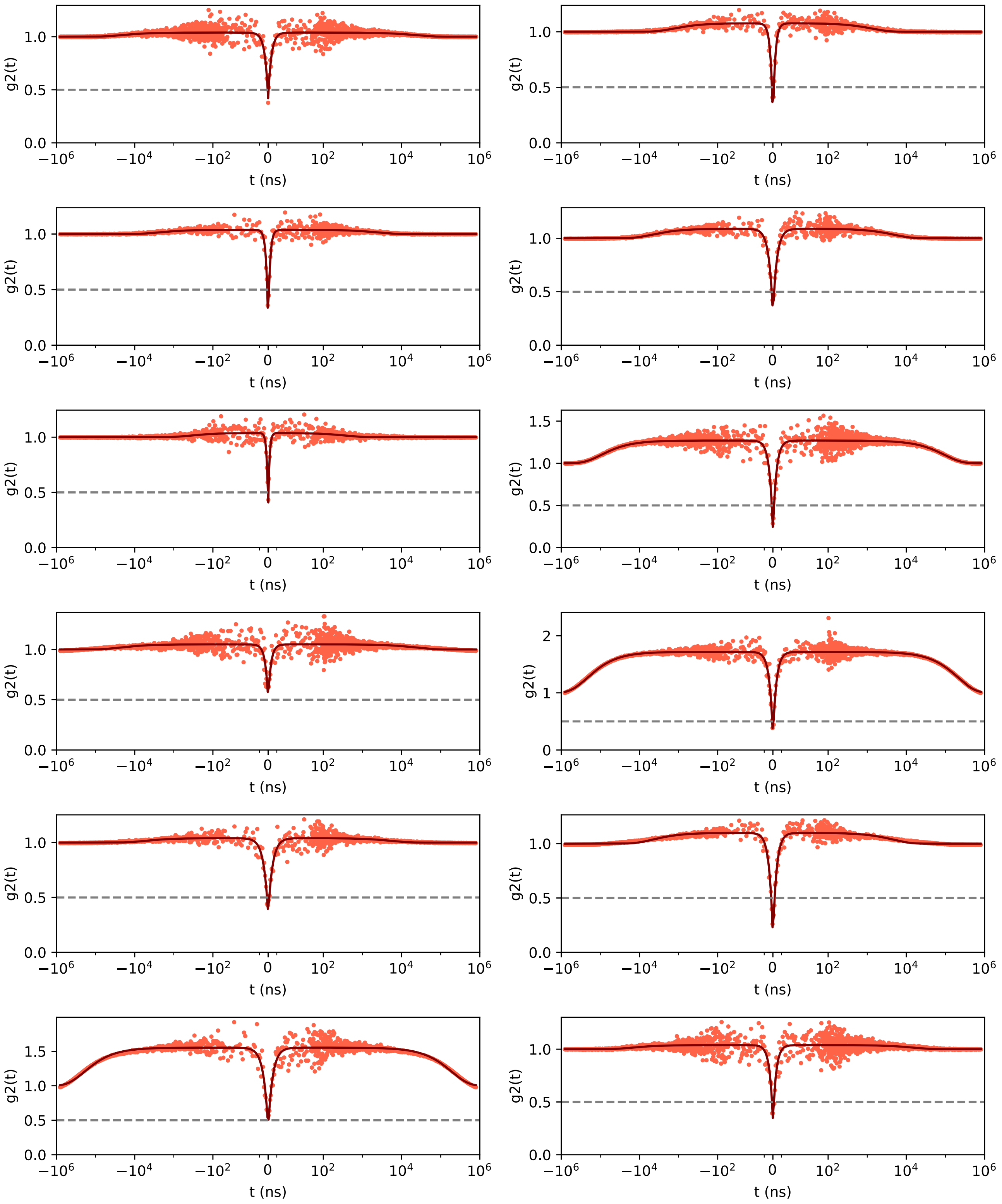

Supplementary Figure 10: $g^{(2)}(\tau)$ measurement for non ODMR-active defects (orange circles), conducted between 0.2 and 0.7 $P_{sat}^{optical}$ laser power, fit to a bi-exponential function (red solid line).

$g^{(2)}(\tau)$ Experimental Background

All experimental $g^{(2)}(\tau)$ data contains sources of noise that increase the value of $g^{(2)}(0)$. These include the instrument response function (IRF) that accounts for timing jitter of the APDs and time correlator[1], the finite bin size[2], and the background fluorescence[3,4]. We can estimate the effect of the IRF and finite bin size from the nominal time jitter specified for the APD used, and from the bin size chosen in data postprocessing. Their effect on the value of $g^{(2)}(0)$ can be simulated with a window function for an ideal single photon emitter where nominally $g^{(2)}(0) = 0$. The background fluorescence needs to be measured experimentally in the proximity of the emitter.

For our defects, the background fluorescence was small (<0.5 kcps) compared to the defect count rate (20-50 kcps), therefore the main source of noise is the IRF and binning. As Supplementary Figure 11 shows, an ideal single-photon source with $g^{(2)}(0) = 0$ is typically not achieved experimentally. The $g^{(2)}(\tau)$ data presented in the main text and Supplementary have not been background corrected as we prefer to show the raw data.

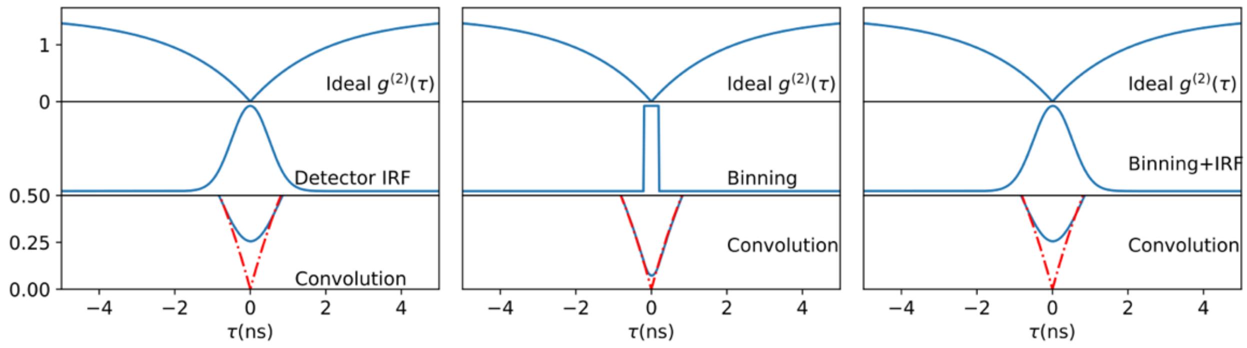

Supplementary Figure 11: Influence of IRF (left panel) and binning (middle) on an ideal $g^{(2)}(\tau)$ function. The right panel shows the effect of both sources of noise on the $g^{(2)}(\tau)$ function.

Bunching Timescale Analysis

Bunching and antibunching rates and amplitudes, obtained from triexponential fits to the $g^{(2)}(\tau)$ data for the ODMR-active and inactive defects, displayed as a function of optical pumping parameter, s, where $s = P_{laser}/P_{sat}^{opt}$. Data for $\tau_{b(additional)}$ show large error on the rates for no-ODMR defects.

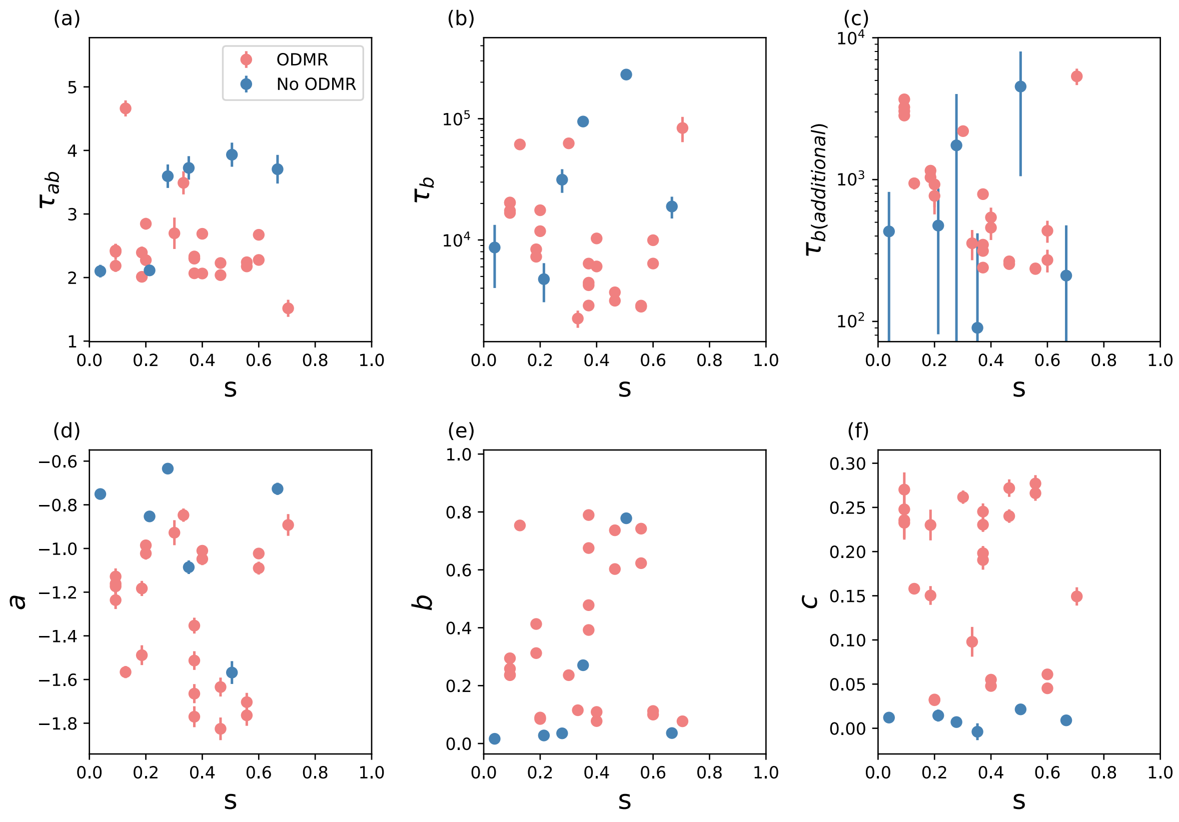

Supplementary Figure 12: Bunching and antibunching rates and amplitudes from defect $g^{(2)}(\tau)$ data. (a)- (c) Antibunching (a), bunching (b) and (c) timescales and corresponding fit amplitudes (d-f).

d) Kinetic Model

For three of our defects we recorded a series of $g^{(2)}(\tau)$ measurements as a function of laser power. The rate constants we determine from the tri-exponential fits to this data enable us to fit our data to a series of rate equations corresponding to a simple three-level kinetic model. We then use this model to explain the ODMR behaviour of our defects. We model our analysis on the approach used by Exarhos et al.[4].

The level structure for this model is shown in the figure below. The model includes a spin-triplet ground (GS) and excited state (ES) and a spin-singlet shelving state, consistent with models previously proposed for hBN spin defects[4,5]. The *ms* = +1 and *ms* = -1 spin sublevels are treated as one state (at zero magnetic field) in the model to simplify computation. The radiative transitions (k_{54} and k_{21}) are spin-preserving and have spin- and laser power-independent rates. The transition rate from GS to ES (k_{12}) is linearly dependent on laser power. Intersystem crossing rates, (k_{23} and k_{53}), and de-shelving rates (k_{34} and k_{31}), are spin-dependent. We find that the intersystem crossing and de-shelving rates must be laser-power dependent to model our $g^{(2)}(\tau)$ data well.

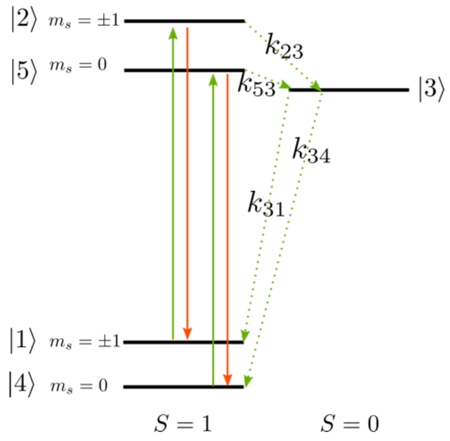

Supplementary Figure 13: Proposed energy level diagram for the hBN defects. Arrows in green indicate transitions that linearly depend on optical excitation power.

The evolution of the excited state population in this model is obtained by solving numerically the rate equation corresponding to the level structure in Supplementary Figure13:

$$\frac{dn_i}{dt} = \sum_j k_{ij} n_j - \sum_m k_{mi} n_i \equiv (\Lambda n)_i, \qquad \text{(Eq. 4)}$$

where n_i is the population in state i, k_{ij} and k_{mi} describe rate of population transfer to and from state j and i. Λ is the rate matrix and is,

$$\Lambda = \begin{bmatrix} -k_{12}s - k_{MW} & k_{21} & k_{31}s & k_{MW} & 0 \\ k_{12}s & -k_{21} - k_{23}s & k_{32}s & 0 & 0 \\ 0 & k_{23}s & -(k_{34} + k_{31} + k_{32} + k_{35})s & 0 & k_{53}s \\ k_{MW} & 0 & k_{34}s & -k_{45}s - k_{MW} & k_{54} \\ 0 & 0 & k_{35}s & k_{45}s & -k_{54} - k_{53}s \end{bmatrix}$$

(Eq. 5)

for our proposed level structure. The rate k_{MW} is a phenomenological constant added to describe the effect of MW-induced spin transitions and is set to zero unless the ODMR contrast is modelled.

This differential equation gives a solution of,

$$n(t) = \sum_i A_i\, e^{-\lambda_i t} u_i \qquad \text{(Eq. 6)}$$

where u_i is the eigenvector of Λ corresponding to eigenvalue λ_i and A_i is a coefficient dependent on the ground state population. The solution to this equation gives the bunching and antibunching amplitudes and rates, allowing comparison with experimental data.

The initial condition for this model corresponds to the ground state configuration following emission of a photon. In the case where the ground state is a singlet ($S = 0$, and spin multiplicity of one), $n_{GS}(t = 0) = 1$. When the ground state is a triplet ($S = 1$, spin multiplicity of three), continuous optical pumping may partially polarize the ground state, such that $n_i(t = 0) \neq n_i(t \to \infty)$ even for $i \in GS$. It is important to incorporate the ground spin polarisation to the model.

Upon measurement of a photon, it can be inferred that the system has decayed from an excited state j to a ground state i. The probability of the emitter in ground state i given a photon has been detected is,

$$n_i(t = 0) = P(i|\text{photon}) \qquad \text{(Eq. 7)}$$

$$= \sum_{j \in ES} P(i|j) P(j|\text{photon}) \qquad \text{(Eq. 8)}$$

$$= \sum_{j \in ES} \frac{k_{ij}}{(\sum_{m \in GS} k_{mj})} \frac{n_j(t \to \infty)}{[\sum_{k \in ES} n_k(t \to \infty)]}, \qquad \text{(Eq. 9)}$$

Where the first factor describes the branching ratio – the proportion of decay into GS i over decay into all ground states from ES j – and the second factor describes the probably the system is in excited state j.

Using this initial condition, Eq.6 is solved by finding the coefficients A_i. $g^{(2)}$ is predicted to be,

$$g^{(2)}(\tau) = \frac{\sum_{i,j} k_{ij} n_i(\tau)}{\sum_{i,j} k_{ij} n_i(\tau \to \infty)} \qquad \text{(Eq. 10)}$$

where $i \in ES, j \in GS$.

<u>Laser Power Dependence</u>

Using this method, the $g^{(2)}(\tau)$ spectrum can be computed and compared with experimental data. We carry out a detailed study on three defects to verify the laser power dependence of $g^{(2)}(\tau)$. In this study the experimental data is fitted with a theoretical model that accounts for the IRF,

$$g^{(2)}_{exp}(\tau) = 1 - \frac{1}{2} a e^{\gamma_1^2 \sigma^2} \left[[1 + \text{erf}(\alpha)] e^{-\gamma_1 \tau} + [1 - \text{erf}(\beta)] e^{+\gamma_1 \tau} \right] + b e^{-\gamma_2 \tau} + c e^{-\gamma_3 \tau}, \qquad \text{(Eq. 11)}$$

Where a, b, c are the antibunching, first bunching and second bunching amplitudes. $\gamma_1, \gamma_2, \gamma_3$ are the corresponding rates. These are fitting parameters. $\alpha, \beta = \frac{(\tau - \gamma_1 \sigma^2)}{\sqrt{2}\sigma}$ are computed from the width of the IRF, σ.Finally, the transition rates from the three-level model (6 in total) are tuned manually to achieve

a set of rates that allow all 6 parameters in the $g^{(2)}(\tau)$ model function to fit the experimental dependence on laser power. In the figure below the fits of the model are shown alongside the antibunching and bunching rates and amplitudes measured for defect C, as a function of optical pumping parameter s, the ratio of excitation laser power over saturation power ($s = P/P_{sat}^{opt}$).

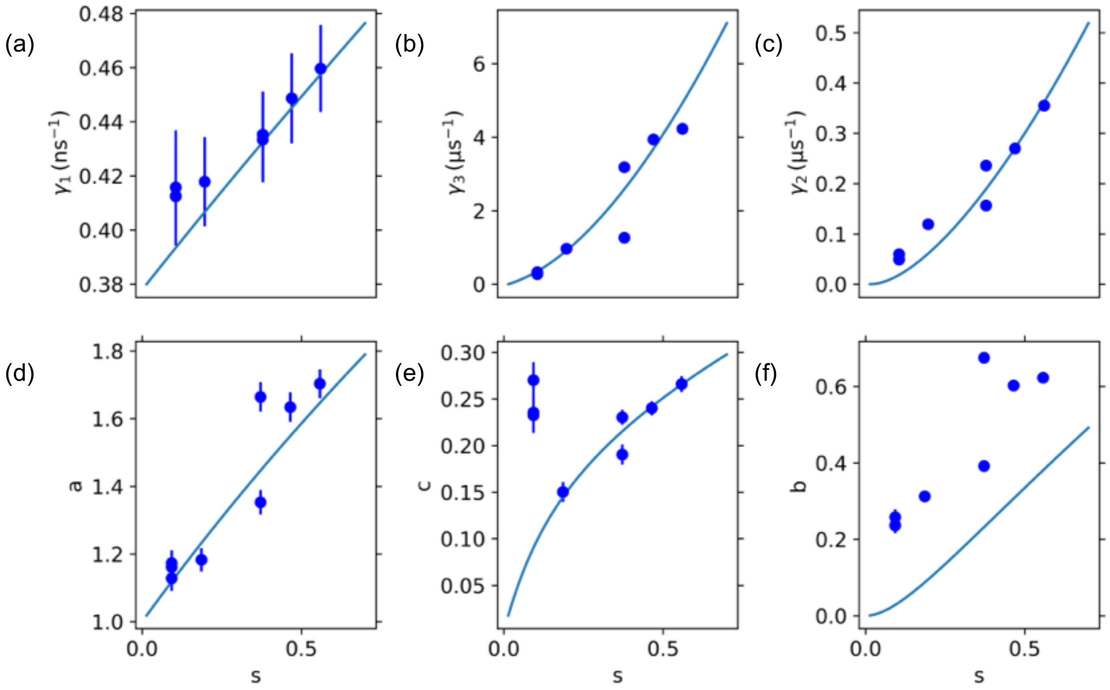

Supplementary Figure 14: Antibunching (a) and bunching (b and c) rates and amplitudes (d-f) for defect C, determined from the laser-power dependent $g^{(2)}(\tau)$ data, fitted with results from the kinetic model. The data has been IRF background corrected.

The fitted rates for defect C are in Supplementary Table 2. These rates are independent of s (Eq. 5).

Supplementary Table 2: Transition rates determined for defect C.

Radiative Transitions		ISC Rates	
$k_{21} = k_{54}$	0.38 ns^{-1}	k_{23}	69.5 μs^{-1}
$k_{12} = k_{45}$	0.076 ns^{-1}	k_{53}	3.47 μs^{-1}
		k_{34}	421 ms^{-1}
		k_{31}	2.25 μs^{-1}

By tuning the optical transition rates, this model explains features including the laser power dependence of $g^{(2)}(\tau)$ data (i), the presence of positive or negative ODMR contrast and the presence of ODMR in only a subset of emitters (ii), as we show in the next section.

(i.) ODMR Contrast as a function of laser power

In Supplementary Figure 15 the experimental ODMR contrast as a function of optical excitation (s) for defect C is shown with the fit from the rate model.

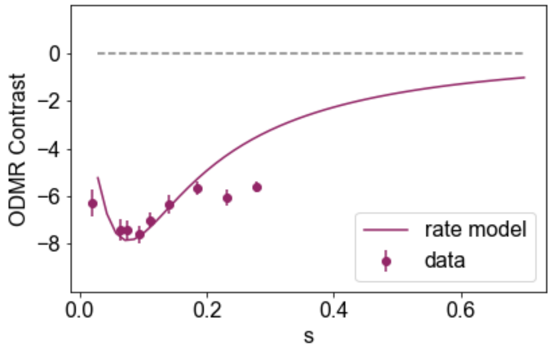

Supplementary Figure 15: ODMR contrast for defect C measured as different optical pumping rates (s = P/P_{sat}^{opt}), with the fit to the rate model.

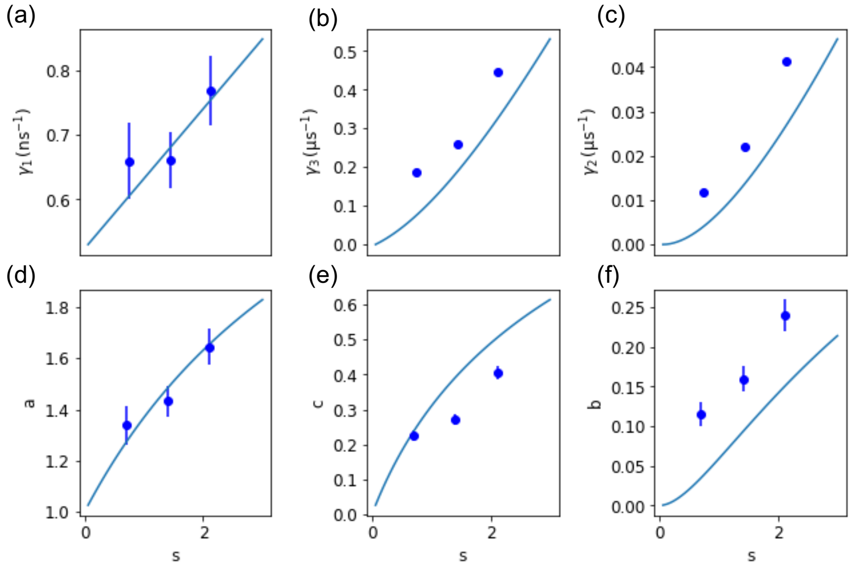

Supplementary Figure 16: Antibunching (a) and bunching (b and c) rates and amplitudes (d-f) for defect A, determined from the laser-power dependent $g^{(2)}(\tau)$ data, fitted with results from the kinetic model. The data has been IRF background corrected.

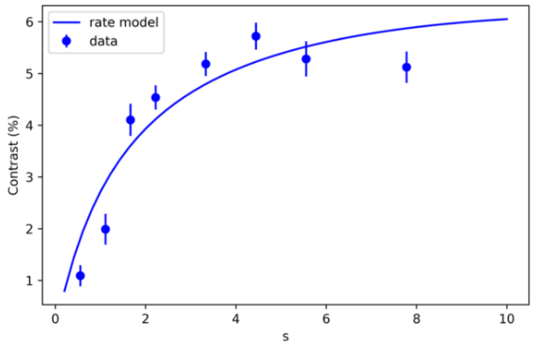

Supplementary Figure 17: ODMR contrast for defect A measured as different optical pumping rates (s = P/P_{sat}^{opt}), with the fit to the rate model.

(ii.) Sign and Magnitude of ODMR Contrast

We find that the ODMR contrast and sign predicted by the model is highly sensitive to the balance of transition rates. In the main text we present the wide range of bunching rates measured for our defects that do and do not show ODMR. Below we give evidence for the tunability of these rates in hBN defects leading to the positive and negative ODMR that we observe.

We use the model to compare the effect on the ODMR contrast when the shelving and de-shelving rates are tuned. In Supplementary Figure 18 we show the ODMR contrast, when the ratio of k_{23}/k_{53}, the two intersystem rates that convert population from the excited state to the metastable state (see fig inset), is varied between 0 and 100. In this simulation the two de-shelving rates (k_{31} and k_{34}) are the same. We can see that ODMR in this case is always negative. However, when the de-shelving rates are asymmetrical, that is k_{31} is greater than k_{34}, the contrast can be positive or negative depending on k_{23}/k_{53}.

This model also shows how defects do not show ODMR when the shelving and de-shelving rates are symmetrical.

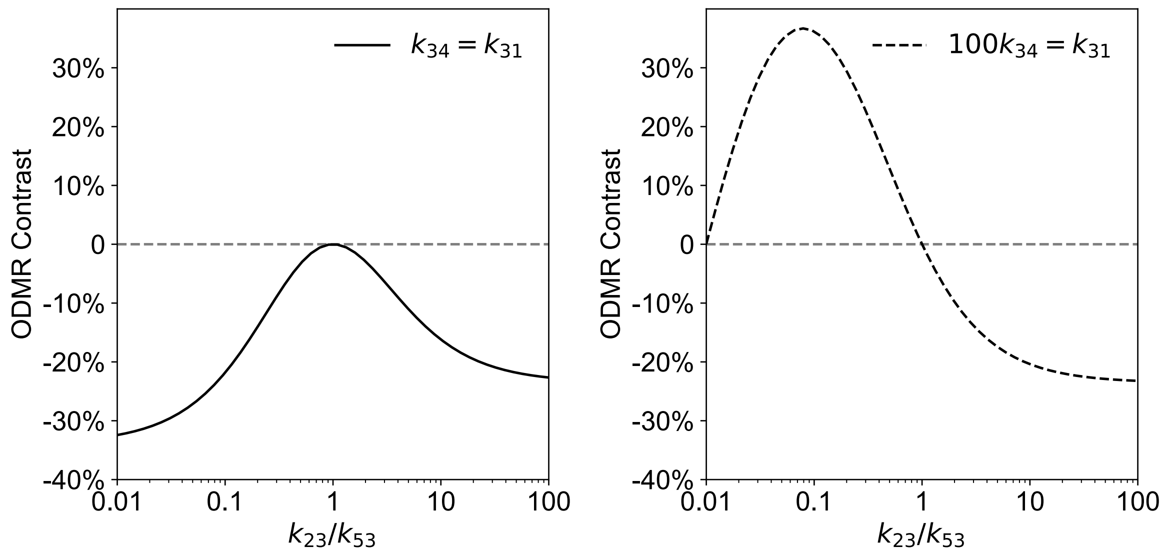

Supplementary Figure 18: ODMR contrast predicted by the rate model determined as a function of the intersystem crossing and de-shelving rates.

2. ODMR of single defects

a) Determination of saturation conditions: microwave power

In the following analysis we show the microwave-power saturation ODMR measurements that were performed as a function of magnetic field to account for the frequency-dependent microwave transmission of our microstrip.

We measure the ODMR with 2-MHz microwave frequency steps and present the data as an average of the two nearest data points. We then fit the ODMR signal to a single Lorentzian with the following function,

$$C = \frac{A\gamma^2}{(\nu-\nu_0)^2+\gamma^2} + y_0 \quad \text{(Eq. 12)}$$

where C is ODMR contrast, A is the amplitude, γ is the half width half maximum, ν_0 is the central frequency and y_0 is the y offset. To fit the ODMR spectra we first remove a static background of 0.2% which is an experimental offset present in all our measurements.

Supplementary Figure 19 shows the ODMR contrast determined from a single Lorentzian fit (Eq. 12) to the ODMR central frequency, for defect A at a range of input microwave powers and magnetic field strengths, at 100-μW laser power. The data is fit to the saturation function (Eq. 1).

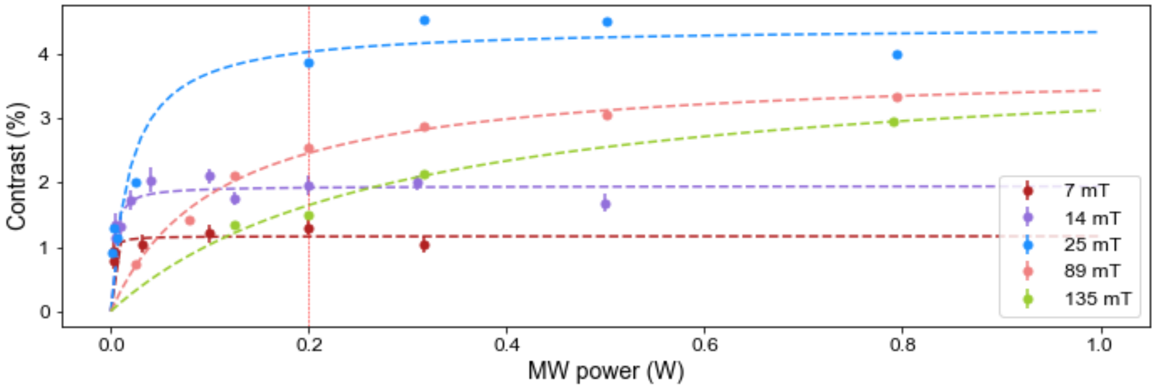

Supplementary Figure 19: The ODMR contrast, at five different magnetic field strengths, as a function of input microwave power (W). The saturation fits (Eq. 1) give a saturation ODMR contrast of 1.2(1)%, 1.9(1)%, 4.4(3)%, 3.8(1)% and 4.0(4)% for 7 mT, 14 mT, 25 mT, 89 mT and 135 mT, respectively.

Fits to the data show that saturation occurs at different values of the microwave power for different microwave frequencies. At 135 mT $10P_{sat}^{microwave}$ corresponds to 3-W input to the microstrip, a power at which we experience troublesome heating and sample drift. For this reason, we chose a fixed microwave power (0.2 W) for the measurements in Fig. 2 of the main text.

Defects A- G

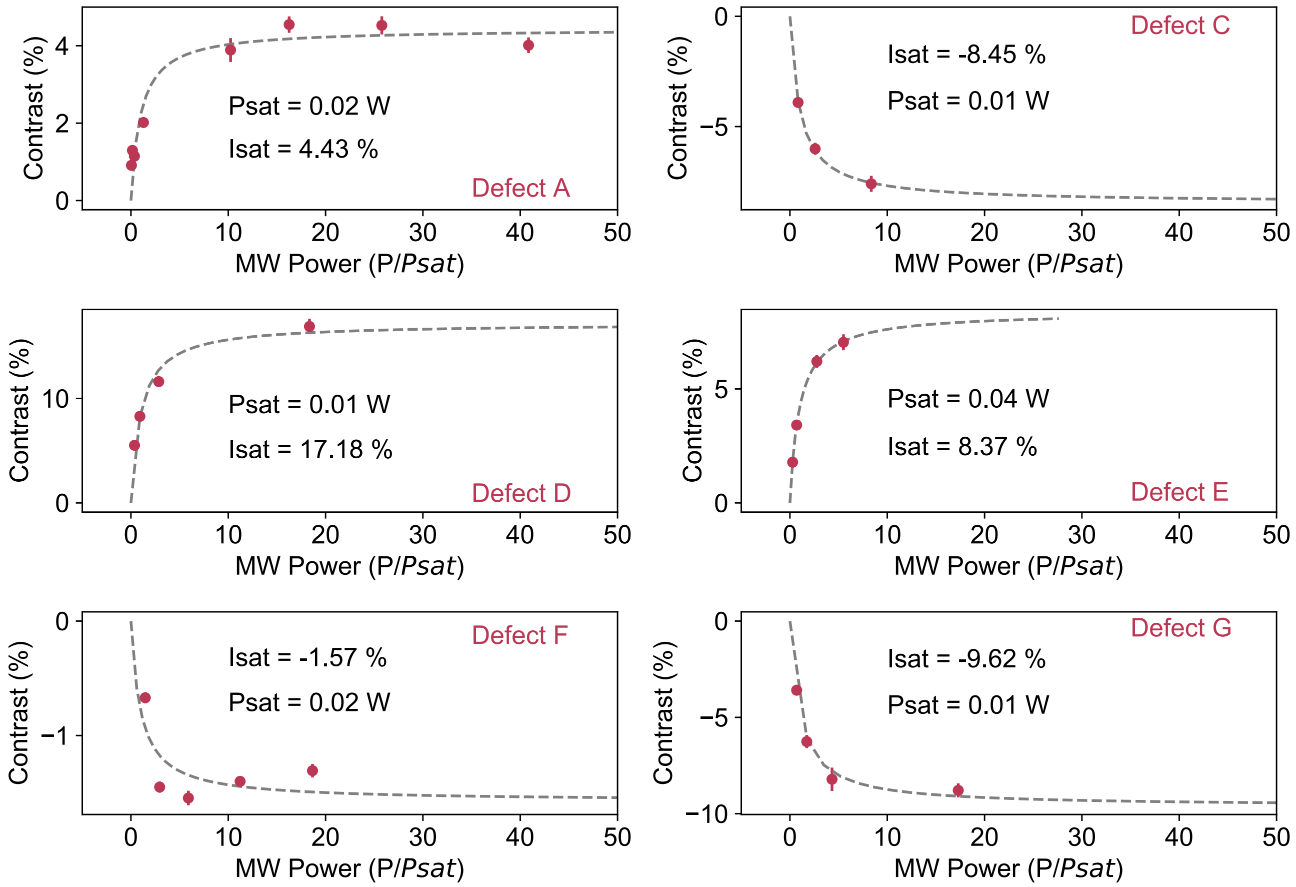

Supplementary Figure 20: ODMR contrast as a function of input MW power for defects A-G. I_{sat}^{MW} and P_{sat}^{MW} are noted in the inset. The x axis is normalised for P_{sat}^{MW}. MW saturation was not measured for defect B, but spectrum in the main text Figure 4 is measured at 0.01W.

b) Analysis of functions fit to model ODMR lineshape

Here we compare four distributions to model the lineshape of the ODMR spectra for defects A, B and K. The measurements were all recorded at $P_{sat}^{microwave}$ and an in-plane magnetic field.

For the Gaussian function we use the following equation,

$$C = A\, e^{\left(\frac{\nu-\nu_0}{2\sigma}\right)^2} + y_0 \qquad \text{(Eq. 13)}$$

where A is the amplitude, ν_0 is the central frequency, σ is the variance and y_0 is the y offset.

For the Voigt function we use the following equation,

$$C = B\, \frac{1}{\sigma\sqrt{2\Pi}} e^{\left(\frac{\nu-\nu_0}{2\sigma}\right)^2} + A\frac{\gamma^2}{(\nu-\nu_0)^2+\gamma^2} \qquad \text{(Eq. 14)}$$

Where C is the contrast, B is the Gaussian amplitude, ν_0 is the ODMR central frequency, σ is the Gaussian variance and A is the Lorentzian amplitude and γ is the Lorentzian half width half max. For the Lorentzian we use Eq. 4 above. For the double Lorentzian we use two Lorentzians that are summed together to model the lineshape.

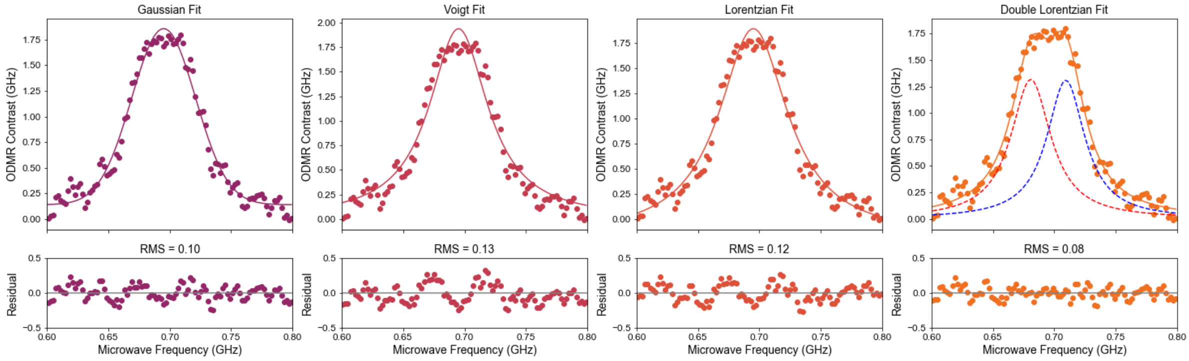

Supplementary Figure 21: Defect A, in-plane 25 mT, $P_{sat}^{microwave}$ showing a double Lorentzian splitting of 29(2) MHz, fit to Gaussian, Voigt, single Lorentzian and Doublet Lorentzian functions. The root mean squared of the residual is noted above the residual plot.

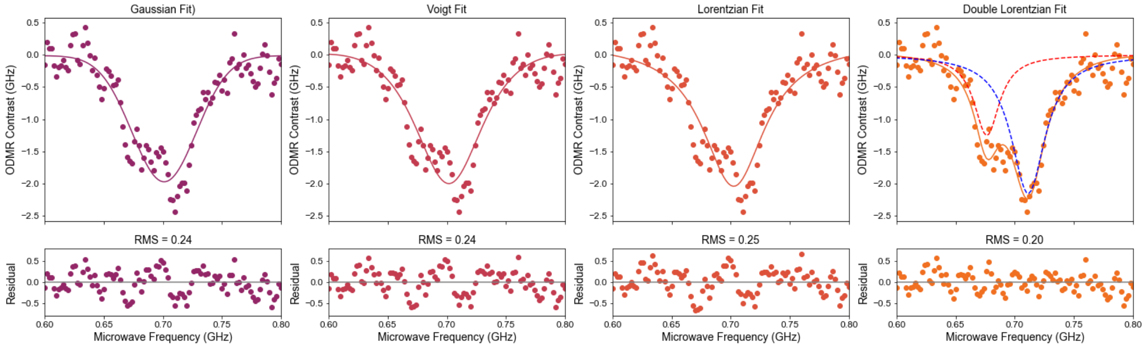

Supplementary Figure 22: Defect B, in-plane 25 mT, showing a double Lorentzian splitting of 35(2) MHz, fit to Gaussian, Voigt, single Lorentzian and Doublet Lorentzian functions. The root mean squared of the residual is noted above the residual plot.

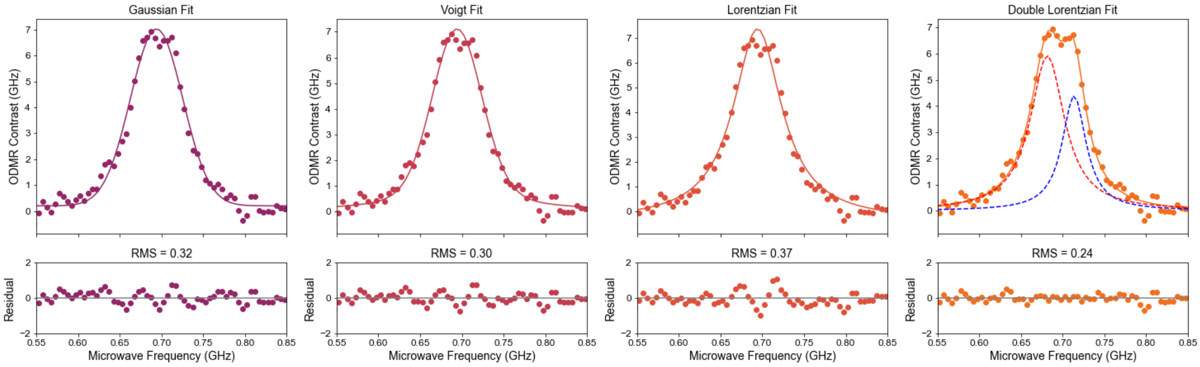

Supplementary Figure 23: Defect D, in-plane 25 mT, P_{sat}^{MW} , showing a double Lorentzian splitting of 32(2) MHz, fit to Gaussian, Voigt, single Lorentzian and Doublet Lorentzian functions. The root mean squared of the residual is noted above the residual plot.

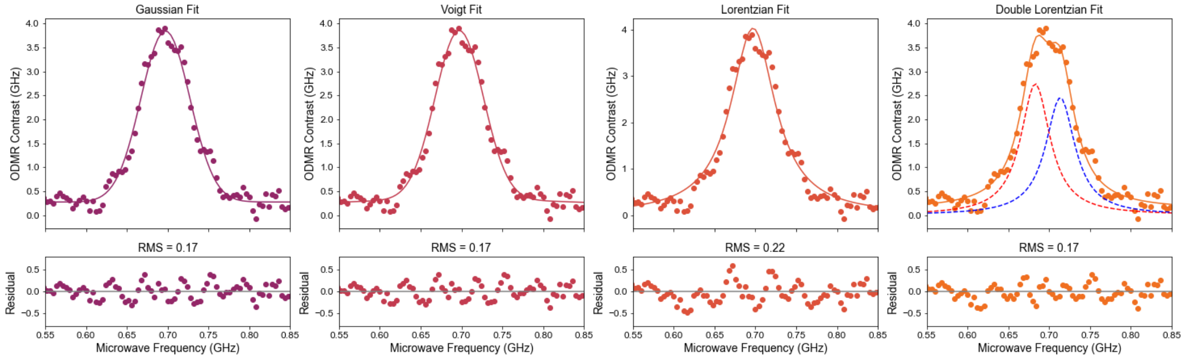

Supplementary Figure 24: Defect E, in-plane 25 mT, 0.7 P_{sat}^{MW}, showing a double Lorentzian splitting of 30(2) MHz, fit to Gaussian, Voigt, single Lorentzian and Doublet Lorentzian functions. The root mean squared of the residual is noted above the residual plot.

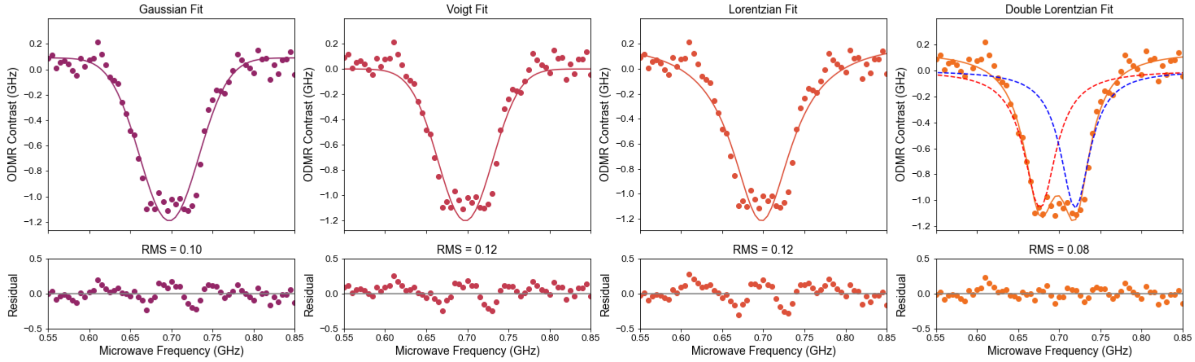

Supplementary Figure 25: Defect I, in-plane 25 mT, $3P_{sat}^{MW}$, showing a double Lorentzian splitting of 42(2) MHz, fit to Gaussian, Voigt, single Lorentzian and Doublet Lorentzian functions. The root mean squared of the residual is noted above the residual plot.

a) <u>PL spectra variation of single defects</u>

The variation in the zero-phonon line position between different defects is shown in Supplementary Figure 26 below for a selectin of ODMR inactive (grey circles) and ODMR active emitters (blue for those showing negative ODMR contrast, red for those showing positive ODMR contrast). For some defects, the emission profiles are clipped by the 550-nm long-pass filter placed to remove the laser light before detection, and therefore we can only estimate the position of their zero-phonon line (hollow circles). We find no clear correlation between the ZPL position and the presence or sign of ODMR contrast.

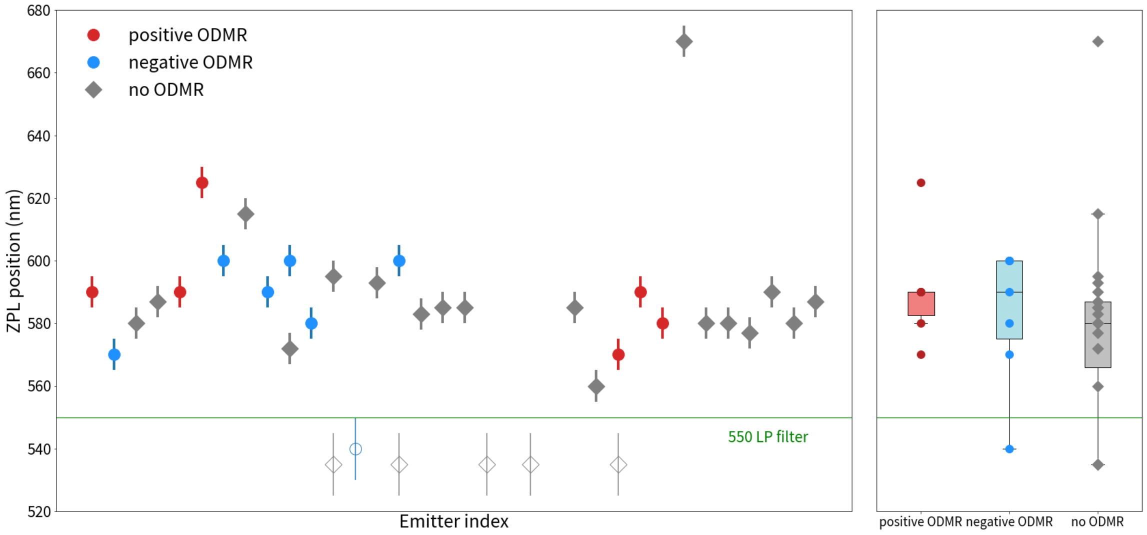

Supplementary Figure 26: Variation in the zero-phonon line position for ODMR-active and inactive defects in the hBN film, as obtained from their photoluminescence spectra with 532-nm excitation.

b) Summary of all ODMR-active defects

From >400 defects scanned, we measure significant ODMR signal (> 1%) on 27 defects. The survey was conducted at a constant in-plane magnetic field of 25 mT and each defect was measured at a range of magnetic field strengths. Not all measurements were performed on all defects and some defects photobleached before a full set of data was measured. The highest saturated ODMR contrast we observe is 35%. (number 18 below).

Supplementary Table 3: ODMR-Active Defect Data

Defect Number	Defect Label in Manuscript	ODMR contrast (saturated) %	Doublet Splitting (MHz)	ZPL (nm) (±10 nm)	P_{sat}^{opt} (uW)	P_{sat}^{MW} (W)	$G^{(2)}(0)$ (fit)
1	A	4.8	29 ± 2	590	70	0.02	0.34
2	B	-6	35 ± 2	570	64		0.35
3	C	-8.5	< 16 ± 2		540	0.01	0.25
4	D	17	32 ± 2		300	0.01	0.45
5	E	8.4	31 ± 4	590	780	0.04	0.39
10	J	-8.5	< 17.5 ± 3	600		0.01	
7	G	-9.6	<12.7 ± 1	600		0.01	
8	H	11.9	< 12 ± 1				
9	I	-1.6	40 ± 2	590		0.02	
6	F	4	< 12.9 ± 1	625			
11	K	-4.1	42 ± 4	580	45	0.03	0.47
12		4.2	36 ± 1		469	0.01	0.14
13		-7	50 ± 6	540	332		0.57
14		-1			500		0.3
15		-2.5	38 ± 8	600			
16		-5	35 ±5				
17		-2	19 ± 7				
18		35					
19		3.5	37 ± 2		440		
20		-2			166		
21		-2			154		
22		-6			386		
23		-3.5					
24		-5					
25		1		570			
26		2	-	590			
27		3		580			

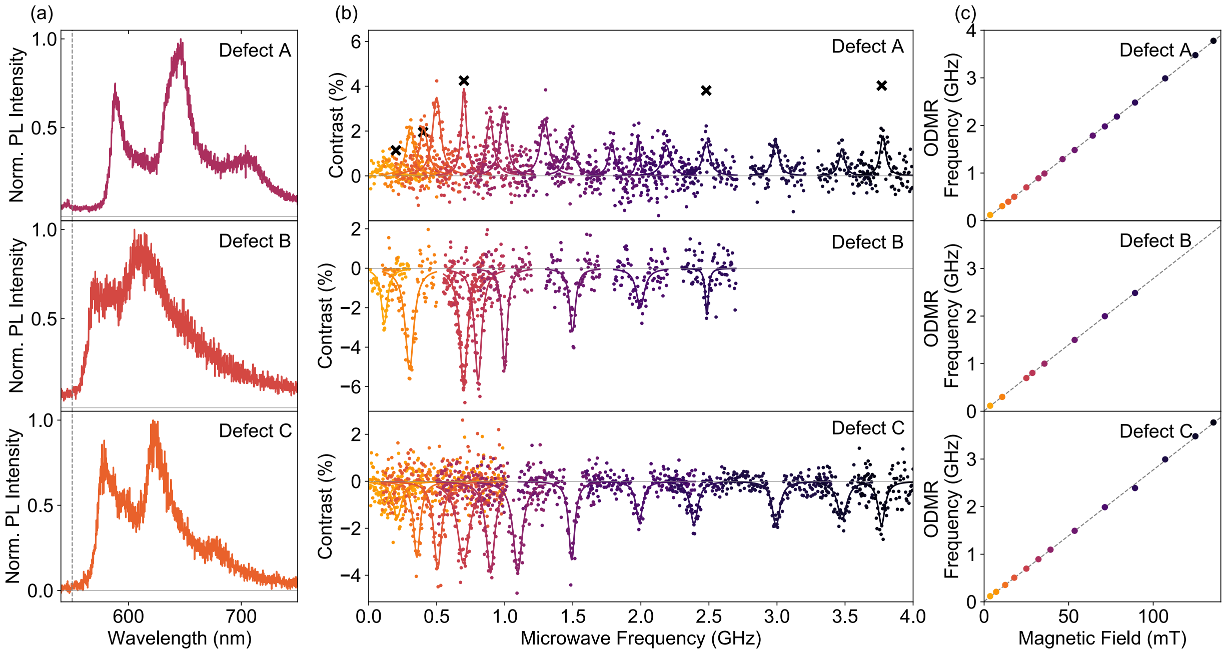

Supplementary Figure 27: Magnetic-Field Dependence of ODMR Resonance. (a) Integrated PL spectra of defects A, B and C under 532-nm optical excitation. (b) ODMR spectra for defects A, B and C as a function of in-plane magnetic field, measured at 0.2W, which is $10P_{sat}^{microwave}$ at 25 mT and $2P_{sat}^{microwave}$ at 89 mT. A constant microwave power was used across the magnetic field range as higher powers cause microwave-induced heating. The black crosses mark the saturated contrast at that magnetic field strength, i.e., 1.1 % and 1.9% at 7 mT and 14 mT, respectively, saturating at ~4% at 25 mT and beyond. (c) ODMR resonance central frequency against magnetic field, fit to a linear function. The g-factors extracted from the linear fits are 1.98(3), 1.97(3) and 1.98 (3) for defects A, B and C, respectively.

3. ODMR of high-density ensemble

a) g-factor calibration

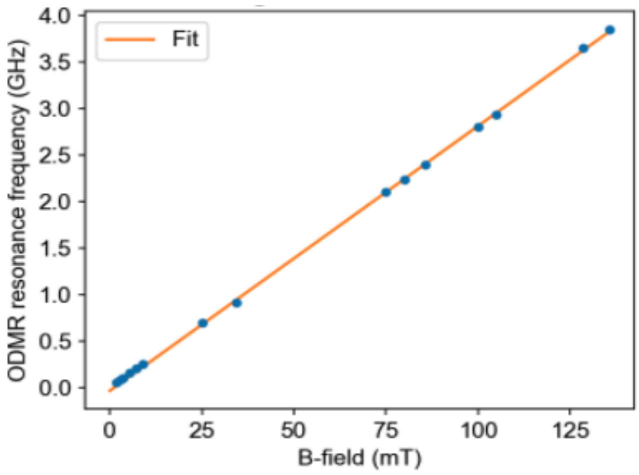

Supplementary Figure 28: Frequency of central peak position against magnetic field strength of the ODMR resonance for the ensemble of spin defects. Each ODMR signal was fit to a Lorentzian (Eq. 3) to extract the central frequency of the resonance. The measurements were conducted with 532 nm laser excitation at 200 μW. A MW power of between 0.9-1 W was used across a loop antenna placed between the hBN film and the microscope objective. We determine a g factor of 2.03(3).

b) ODMR lineshape of high-density ensemble

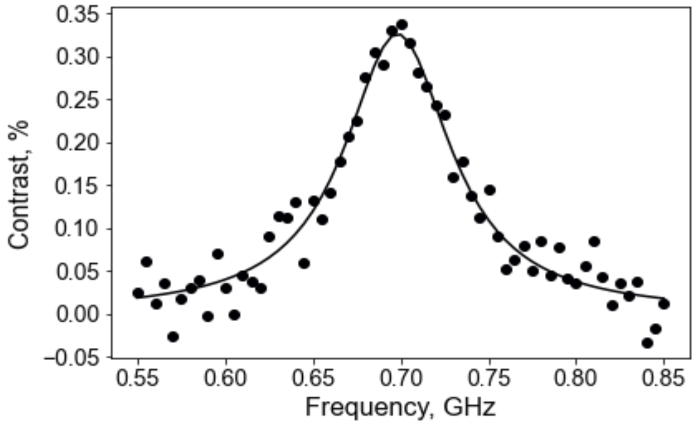

Supplementary Figure 29: CW-ODMR spectrum of the ensemble of spin defects in the h-BN film fit to a Lorentzian. The measurement was conducted at 100 μW laser power and $P_{sat}^{microwave}$ microwave power applied across the microwave microstrip (see detailed in Methods in the main text). The linewidth determined from a single Lorentzian fit is 37(2) MHz.

4. EPR of high-density ensemble

CW-EPR measurements were performed on the ensemble using a Bruker E500 X-band spectrometer with a ER 4122SHQE cavity at a microwave frequency of 9.370 GHz and a microwave power of 2mW. The external magnetic field was modulated at 100 kHz with an amplitude of 0.5 mT and the spectra were recorded as first harmonics of the absorption signal. The device was mounted in the centre of the cavity on a high-purity quartz slide. 532 nm laser light was coupled into the cavity through an optical window and focused on to the device with a spot size of ~2.5 mm diameter. All ESR measurements were carried out at room temperature as a function of the average laser power ranging from 10 mW to 130 mW. They are reported after background subtraction from the sample mount.

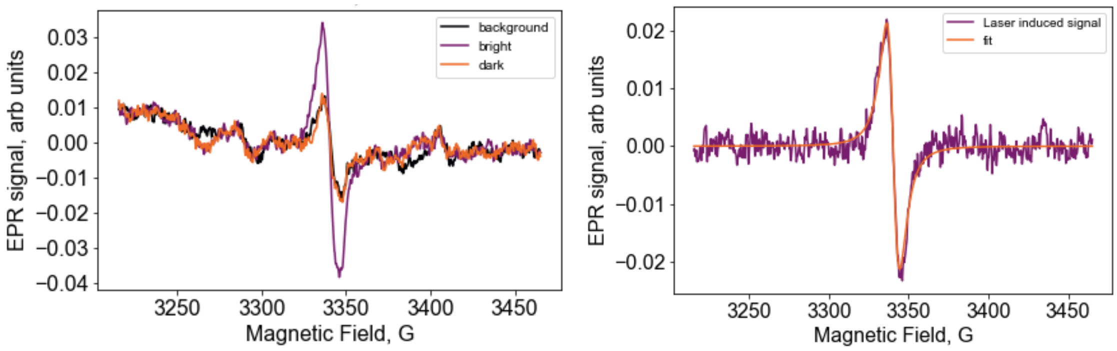

Supplementary Figure 30: (left panel) The room temperature background, dark and light-induced EPR signal for the ensemble, measured using 532 nm laser excitation with a power of 55 mW over an area of 2.5 mm diameter spot on sample. (right panel) The light-induced EPR signal for the ensemble with background and dark signals subtracted. A Lorentzian fit to the linewidth gives 7.1 G (~20 MHz).

5. Spin Hamiltonian for S=1

In the main text we consider two situations to explain the observation of the ODMR lineshape that can display both singlets with 20-30 MHz linewidth, or doublets split by 20-50 MHz: hyperfine coupling and zero-field splitting (ZFS).

To consider how a ZFS model could explain the doublet, we can use the following spin Hamiltonian for a S =1 system[6], similar to NV centres in bulk diamond,

$$H = g\mu_B \mathbf{B}\cdot\mathbf{S} + D\left(S_{z\prime}^2 - \frac{1}{3}S(S+1)\right) + E\left(S_{x\prime}^2 - S_{y\prime}^2\right), \qquad \text{(Eq. 15)}$$

where D and E are the ZFS parameters, B is the magnetic field, and $\boldsymbol{S} = (S_{x\prime}, S_{y\prime}, S_{z\prime})$ is the spin projection operator corresponding to a S=1 system. The Hamiltonian takes this form in the frame spanned by the eigen-vectors $\boldsymbol{x}', \boldsymbol{y}', \boldsymbol{z}'$ of the ZFS tensor, hereafter referred to as the "defect frame". In the case of small zero-field splitting, i.e. ~ 10 MHz (as the g-factor for the defects indicates), the magnetic fields we apply are in the 'high-field limit', i.e $g\mu_B\mathbf{B_z}$ >> D and assuming E~0. Under this condition, the two allowed transitions take place with energies of $|g\mu_B\mathbf{B_z} + D|$ and $|D - g\mu_B\mathbf{B_z}|$), split in energy by 2D (as shown in Figure S30). The splitting of the doublet would remain constant as the magnetic field is reduced until the Zeeman splitting is no longer the dominating interaction when $g\mu_B\mathbf{B_z}/h$ ~ 10MHz, by which time the ODMR contrast has diminished to prevent a clear identification of the low field behaviour.

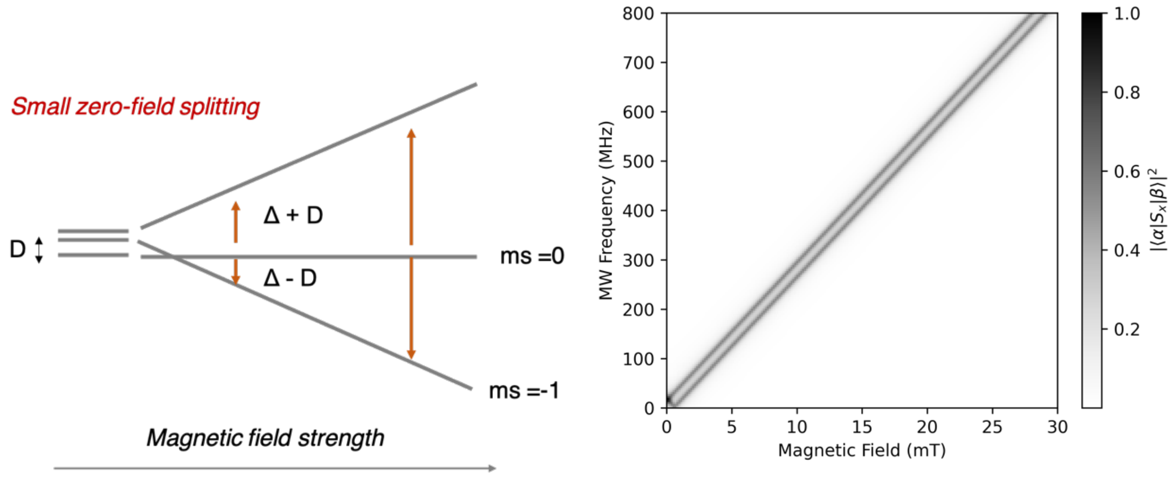

Supplementary Figure 31: left: Schematic showing the Zeeman splitting and microwave transitions expected for a system with low zero-field splitting. Right: the corresponding modelled ODMR transitions (z axis is transition strength) as a function of microwave frequency and magnetic field.

To predict the transition probability of the ground state spin upon RF drive, a drive term is added to Eq. 15,

$$H_D = \mu_B g(\boldsymbol{S}\cdot\boldsymbol{W}_0)\cos\omega_D t, \qquad \text{(Eq. 16)}$$

where $\boldsymbol{W}_0 \cos \omega_D t$ is the AC magnetic field created by RF frequency drive. In the limit of small RF drive amplitude compared to ZFS and Zeeman terms, the driving term can be treated perturbatively and the transition probability per unit time from state i to state f is given the Fermi's Golden Rule[7],

$$\Gamma_{i\rightarrow f} = \frac{2\pi}{\hbar} |\langle i|\mu_B g(\boldsymbol{S}\cdot\boldsymbol{W}_0)|f\rangle|^2 \delta\left(\omega_D - \left|E_i - E_f\right|/\hbar\right), \qquad \text{(Eq. 17)}$$

where $|i\rangle$ and $|f\rangle$, and E_i and E_f are the unperturbed eigenstates and eigen-energies of (Eq. 15). Equations 15 and 17 are solved numerically in the molecular frame. Vectors $\boldsymbol{W}_0$ and $\boldsymbol{B}$ are measured in the lab frame and are thus first transformed to the defect frame, via a set of intrinsic Euler angles (ϕ, θ, ψ) using the zxz-convention.

When presenting the simulated results in the main text, the δ-function in Eq. 17 is replaced with a Lorentzian profile with a Full Width at Half Maximum (FWHM) of 30 MHz, derived from the single Lorentzian linewidth measured experimentally. This broadening could be due to dephasing, power broadening, or unresolved hyperfine coupling. The quantity,

$$\left|\langle i|\left(\boldsymbol{S}\cdot\frac{\boldsymbol{W}_0}{|\boldsymbol{W}_0|}\right)|f\rangle\right|^2, \qquad \text{(Eq. 18)}$$

is plotted in the colormap in the main text as it is proportional to the transition probability, and sums to one when all transitions are considered.

a) Angular-dependent ODMR measurements for hBN defects.

Magnetic field angular-dependent measurements were conducted on a range of defects by moving the permanent magnet around the sample on a rotation mount. A limited range of angles were available to measure, due to space constraints around the sample. The field position in the lab frame, B_x, B_y and B_z, were measured using a Teslameter magnetic field probe in the focal spot position. The fits to the following data are from the S=1 model, with *D*= 25 MHz and *E*= 5 MHz, with the orientation of the symmetry axis of the defect's **D** tensor (termed in caption the 'defect's symmetry axis', which is obtained from the fit to the model), noted for each defect in the caption, and the magnetic field vectors (calibrated) and magnitude (fit to data) in the inset. For some defects measurements were taken at zero magnetic field strength. These are shown here along with model.

Defect A

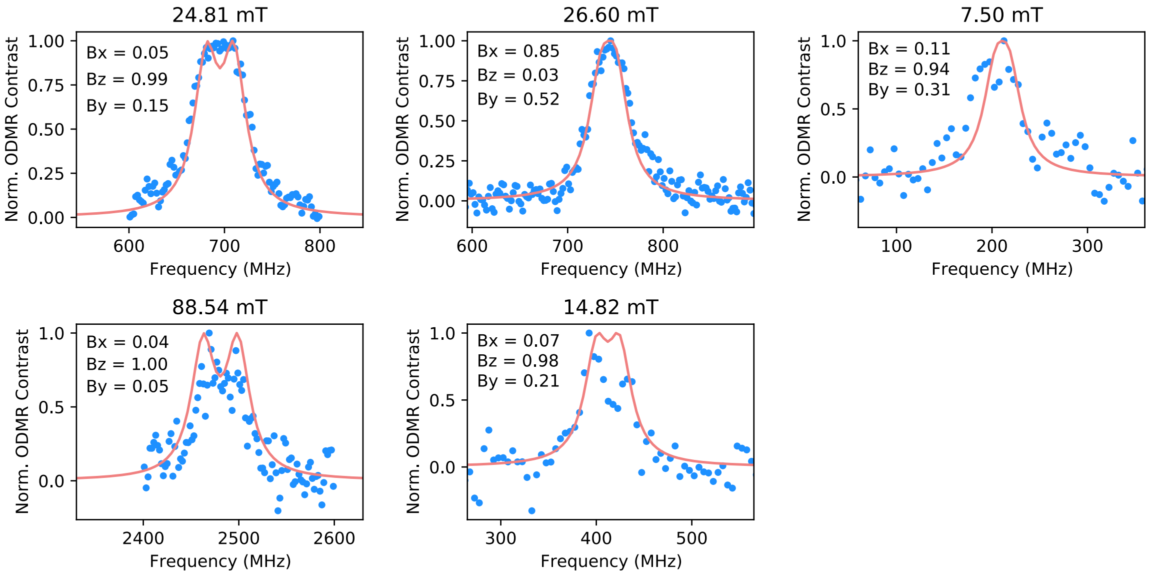

Supplementary Figure 32: ODMR spectra and S=1 model for defect A at a range of magnetic field strengths and orientations. For each measurement, the field strength is given in the title and the orientation in the inset. Orientation of defect symmetry axis: $\phi = 0°$, $\vartheta = -20°$, $\varphi = 0°$.

Defect B

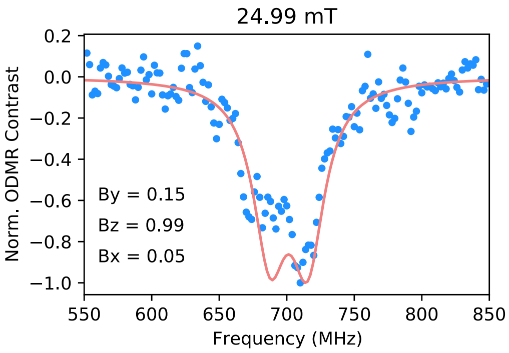

Supplementary Figure 33: ODMR spectra and S=1 model for defect B at 25 mT. The magnetic field orientation is in the inset. Orientation of defect symmetry axis: $\phi = 0°$ $\vartheta = -60°$ $\varphi = 0°$

Defect C

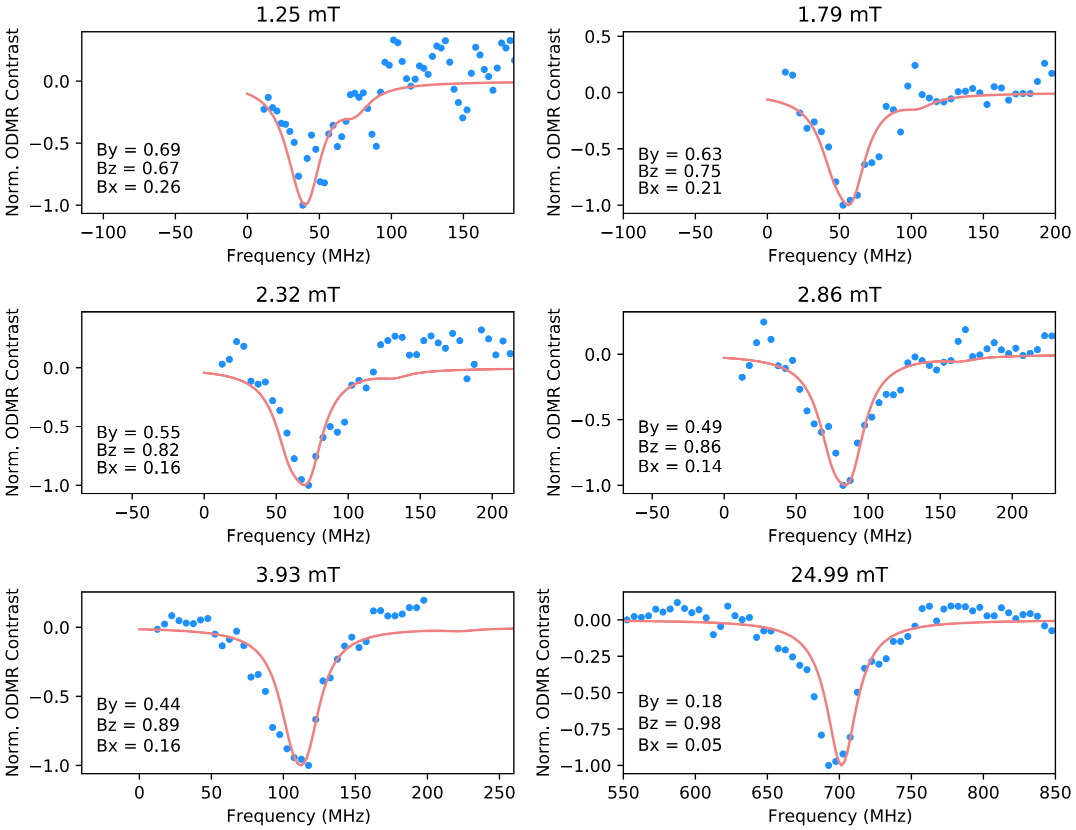

Supplementary Figure 34: ODMR spectra and S=1 model for defect C at a range of magnetic field strengths and orientations. For each measurement, the field strength is given in the title and the orientation in the inset. The magnetic field strength is given in each figure title. Orientation of defect symmetry axis: $\phi = -120° \; \vartheta = -45° \; \varphi = -35°$.

Defect D

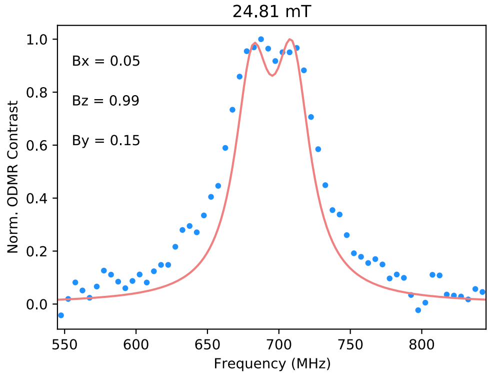

Supplementary Figure 35: ODMR spectra and S=1 model for defect D at a range of magnetic field strengths and orientations. For each measurement, the field strength is given in the title and the orientation in the inset. Orientation of defect symmetry axis: $\phi = 0°, \vartheta = -60°, \varphi = 0°$.

.

Defect E

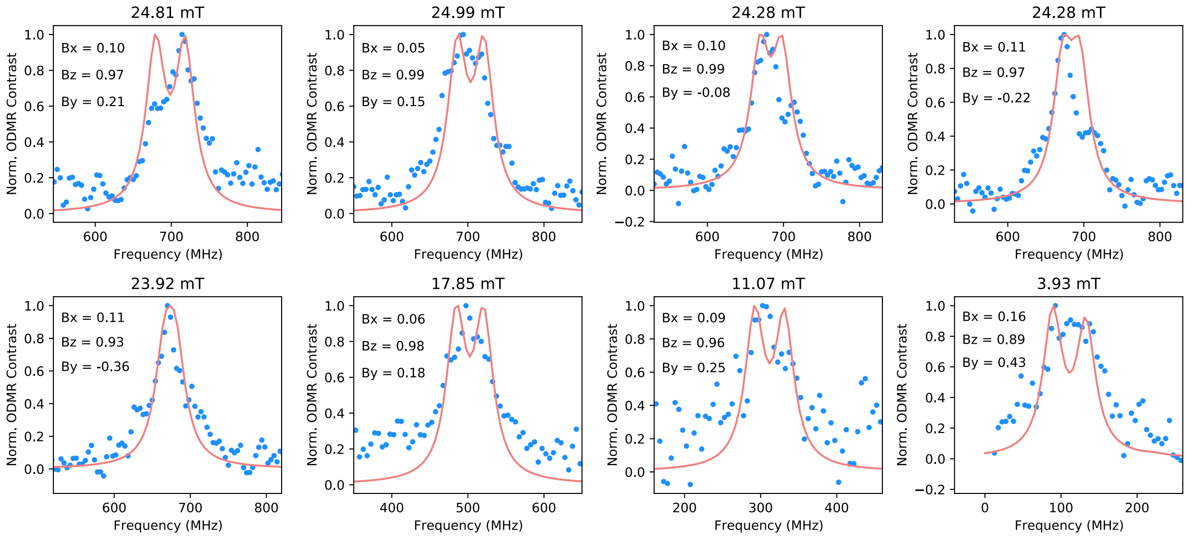

Supplementary Figure 36: ODMR spectra and S=1 model for defect E at a range of magnetic field strengths and orientations. For each measurement, the field strength is given in the title and the orientation in the inset. Orientation of defect symmetry axis: $\phi = 35°, \vartheta = 45°, \varphi = 70°$.

Defect F

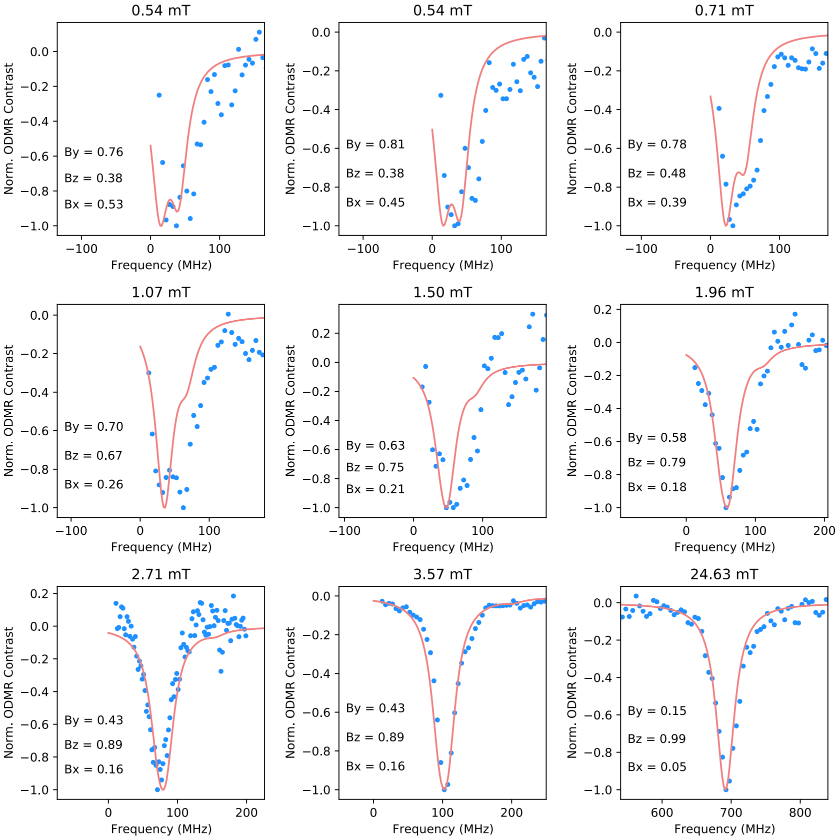

Supplementary Figure 37: ODMR spectra and S=1 model for defect F at a range of magnetic field strengths and orientations. For each measurement, the field strength is given in the title and the orientation in the inset. The orientation of defect symmetry axis: $\phi = -120°, \vartheta = -45°, \varphi = 120°$.

Defect G

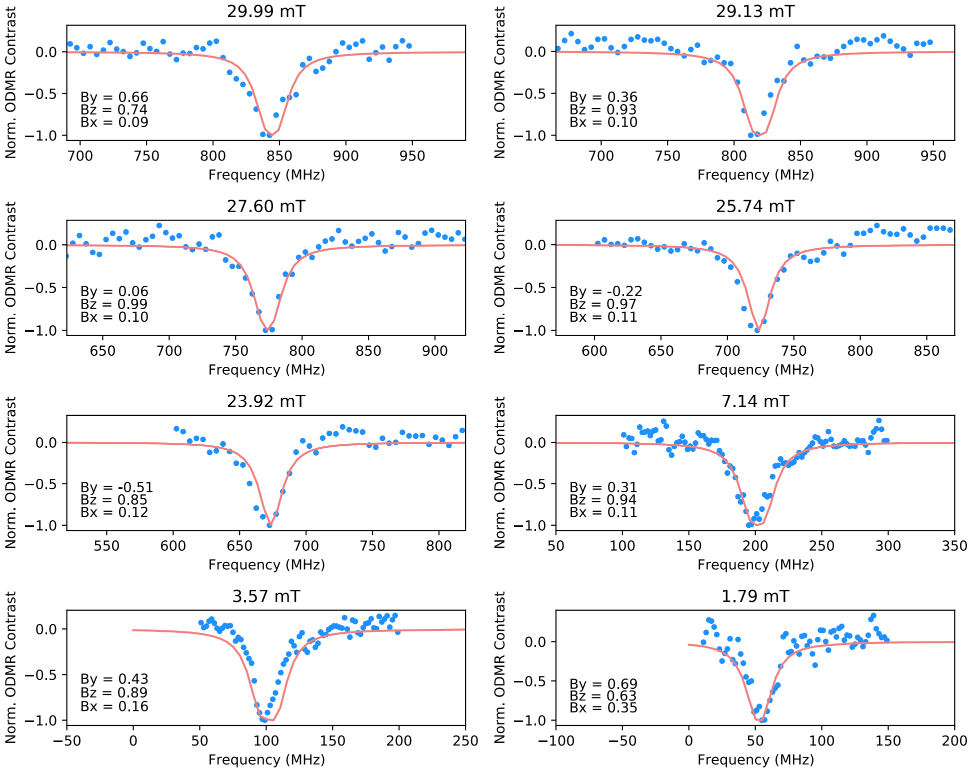

Supplementary Figure 38: ODMR spectra and S=1 model for defect G at a range of magnetic field strengths and orientations. For each measurement, the field strength is given in the title and the orientation in the inset. Orientation of defect symmetry axis:$\phi = -120°$ $\vartheta = 45°, \varphi = 120°$.

Defect H

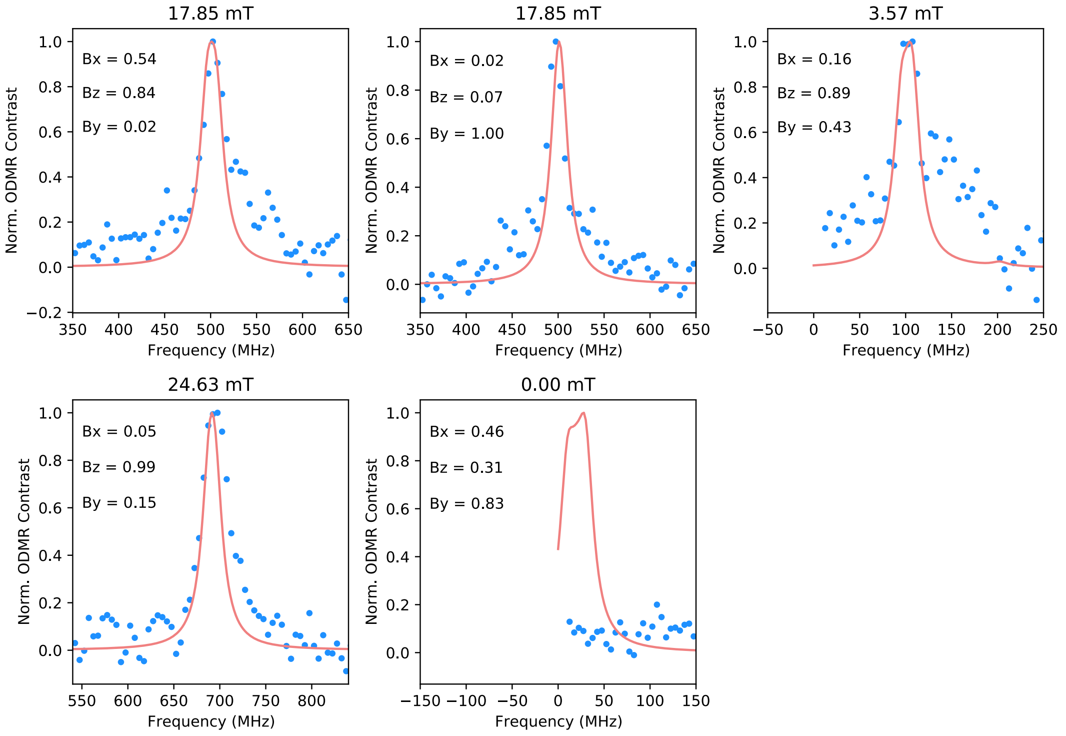

Supplementary Figure 39: ODMR spectra and S=1 model for defect H at a range of magnetic field strengths and orientations. For each measurement, the field strength is given in the title and the orientation in the inset. Orientation of defect symmetry axis: $\phi = -120° \ \vartheta = 45° \ \varphi = 120°$. No signal was measured at zero field (last panel), so data is normalised to max signal*0.2, in line with background of other measurements.

Defect I

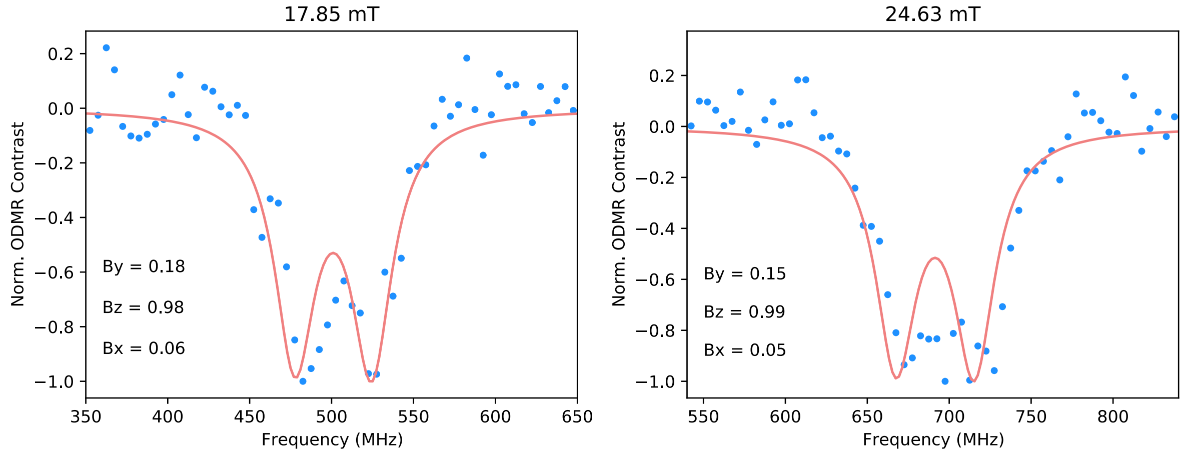

Supplementary Figure 40: ODMR spectra and S=1 model for defect I at a couple of magnetic field strengths and orientations. For each measurement, the field strength is given in the title and the orientation in the inset. Orientation of defect symmetry axis: $\phi = 0° \ \vartheta = 0° \ \varphi = 0°$.

Defect J

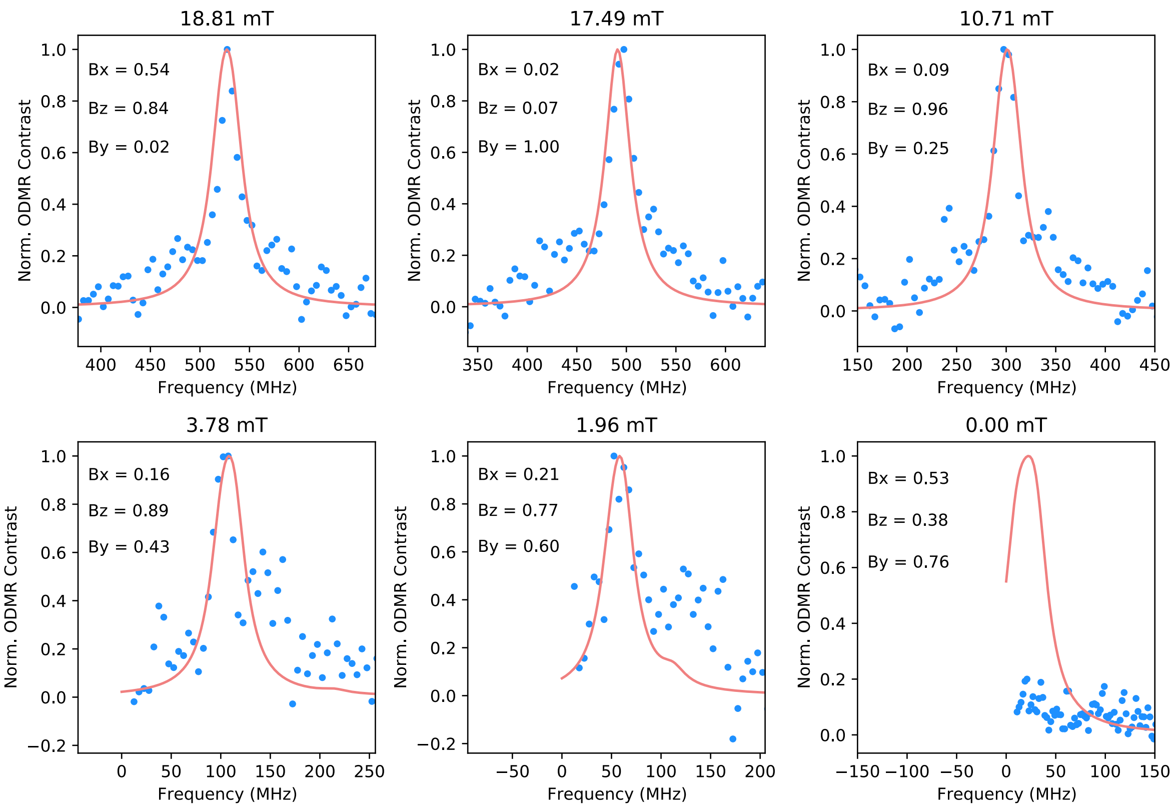

Supplementary Figure 41: ODMR spectra and S=1 model for defect J at a range of magnetic field strengths and orientations. For each measurement, the field strength is given in the title and the orientation in the inset. Orientation of defect symmetry axis: $\phi = -120° \vartheta = -45° \varphi = 120°$. No signal was measured at zero field (last panel), so data is normalised to max signal x 0.2, in line with background of other measurements.

6.) Supplementary References

[1] Bommer, A., and Becher, C. New insights into nonclassical light emission from defects in multi-layer hexagonal boron nitride. *Nanophotonics*, **8**, 11, 2041–2048, (2019).
[2] Thorn, JJ., *et al*., Observing the quantum behavior of light in an undergraduate laboratory. *American Journal of Physics*, **72**, 9, 1210–1219, (2004).
[3] Berthel, M., *et al*., Photophysics of single nitrogen-vacancy centers in diamond nanocrystals. *Physical Review B*, 91, 3, 035308, (2015).
[4] Exarhos, A.L. *et al.* Magnetic-field-dependent quantum emission in hexagonal boron nitride at room temperature. *Nat. Commun*. **10**, 222 (2019).
[5] Chejanovsky, N., *et al.* Single-spin resonance in a van der Waals embedded paramagnetic defect. *Nat. Mater.* **20,** 1079-1984 (2021).
[6] Weil, J. A., & Bolton, J. R. (2007). *Electron paramagnetic resonance: elementary theory and practical applications*. John Wiley & Sons.
[7] Abragam, A. (1961). *The principles of nuclear magnetism* (No. 32). Oxford university press.
